# Berberine Derivative B68 Promotes Tumor Immune Clearance by Dual‐Targeting BMI1 for Senescence Induction and CSN5 for PD‐L1 Degradation

**DOI:** 10.1002/advs.202413122

**Published:** 2024-12-25

**Authors:** Hongmei Hu, Qun Wang, Dianping Yu, Xiaoyu Tao, Mengmeng Guo, Saisai Tian, Qing Zhang, Mengting Xu, Xiangxin Geng, Hongwei Zhang, Hanchi Xu, Linyang Li, Shize Xie, Kaixian Chen, Weiliang Zhu, Xu‐Wen Li, Hanchen Xu, Bo Li, Weidong Zhang, Sanhong Liu

**Affiliations:** ^1^ Shanghai Frontiers Science Center of TCM Chemical Biology Institute of Interdisciplinary Integrative Medicine Research Shanghai University of Traditional Chinese Medicine Shanghai 201203 China; ^2^ State Key Laboratory of Chemical Biology, Drug Discovery and Design Center Shanghai Institute of Materia Medica Chinese Academy of Sciences Shanghai 201203 China; ^3^ University of Chinese Academy of Sciences Beijing 100049 China; ^4^ Department of Phytochemistry School of Pharmacy Second Military Medical University Shanghai 200433 China; ^5^ State Key Laboratory of Drug Research Drug Discovery and Design Center Shanghai Institute of Materia Medica Chinese Academy of Sciences Shanghai 201203 China; ^6^ Institute of Digestive Diseases Longhua Hospital Shanghai University of Traditional Chinese Medicine Shanghai 200032 China; ^7^ Institute of Medicinal Plant Development Chinese Academy of Medical Sciences and Peking Union Medical College Beijing 100193 China

**Keywords:** BMI1, colorectal cancer, CSN5, PD‐L1, tumor cell senescence

## Abstract

Promoting tumor cell senescence arrests the cell cycle of tumor cells and activates the immune system to eliminate these senescent cells, thereby suppressing tumor growth. Nevertheless, PD‐L1 positive senescent tumor cells resist immune clearance and possess the ability to secret various cytokines and inflammatory factors that stimulate the growth of tumor cells. Consequently, drugs capable of both triggering senescence in tumor cells and concurrently diminishing the expression of PD‐L1 to counteract immune evasion are urgently needed. Here, a berberine derivative B68 is developed, which specifically induces tumor cell senescence by targeting BMI1. B68 also involves the degradation of PD‐L1 by targeting CSN5, thereby disrupting the immunosuppressive PD‐1/PD‐L1 interaction and enabling rapid clearance of senescent tumor cells. This approach simultaneously inhibits tumor progression and activates T cell immunity, as evidenced by the robust antitumor response following B68‐induced immunization of senescent cancer cells. Moreover, the synergistic effect of B68 with anti‐CTLA4 therapy further enhances antitumor immunity, and its ability to induce senescence in cancer cells triggers a strong protective response by dendritic and CD8^+^ T cells. These findings provide a scientific basis for developing a new tumor treatment strategy based on senescence induction and lay the foundation for further preclinical research.

## Graphical abstract

B68 uniquely induces tumor cell senescence by directly targeting BMI1, which blocks the cell cycle and inhibits tumor growth, and also targets CSN5 to reduce the expression of PD‐L1, which blocks PD‐1/PD‐L1 immunosuppression, thereby enhancing the ability of the immune system to eliminate senescent tumor cells and exert antitumor effects, providing new research ideas and strategies for the treatment of colorectal cancer.

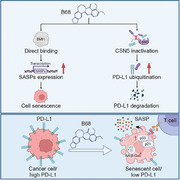

## 1 Introduction

Colorectal cancer (CRC) is the third most common cancer worldwide and the second leading cause of cancer‐related death.^[^ ^1^, ^2^ ^]^ Currently, surgical resection and chemotherapy are the main options for CRC treatment and may improve the 5‐year survival rate of patients.^[^ ^3^, ^4^ ^]^ However, these chemotherapeutic drugs primarily induce tumor cell apoptosis, which does not yield satisfactory therapeutic outcomes and is accompanied by significant drug resistance and toxic side effects.^[^ ^5^, ^6^, ^7^ ^]^ Since the progression of tumors to malignant cancers necessitates overcoming the constraints of senescence, activating the function of tumor suppressor genes or triggering senescence signals could induce senescence in tumor cells.^[^ ^8^, ^9^ ^]^ Furthermore, senescent tumor cells within the body can be eliminated by immune cells or other pathways, thereby effectively suppressing tumor progression.^[^ ^8^, ^10^ ^]^ Thus, inducing senescence in tumor cells may represent a potential therapeutic approach for cancer treatment.

Cancer cells are typically exposed to a variety of known stressors that can trigger senescence, including oncogenic signals, replicative stress, hypoxia, reactive oxygen species, nutrient deprivation, and exposure to cytokines present in the tumor microenvironment, such as TGF‐β.^[^ ^11^, ^12^ ^]^ In addition, cytokines produced by immune cells can also induce senescence within tumors, such as IFNγ produced by Th1 cells. Senescent cancer cells themselves do not promote tumor growth due to their low or nonproliferative capacity. However, they can alter the tumor microenvironment through the senescence‐associated secretory phenotype (SASP).^[^ ^13^, ^14^ ^]^ Notably, the SASP of tumor cells can recruit and activate CD4^+^ and CD8^+^ T cells, thereby inducing antitumor protection.^[^ ^15^ ^]^ Furthermore, senescent cell vaccines may suppress tumor growth by inducing cancer cells to enter a state of senescence and be cleared by the immune system.^[^ ^16^ ^]^ Additionally, in some cases, senescent cell vaccines may help reverse the resistance of tumors to traditional chemotherapy by activating the immune system to clear drug‐resistant tumor cells.^[^ ^17^ ^]^ Therefore, cancer cell senescence has become a new frontier in drug development.

However, senescent cells can also accelerate tumor development by secreting inflammatory cytokines, growth factors and MMPases, which promote tumor aggressiveness, metastatic ability, and angiogenesis, thereby providing them with the nutrients and oxygen needed for growth.^[^ ^18^ ^]^ Furthermore, studies have indicated that slightly senescent cells heterogeneously express the immune checkpoint protein programmed death‐ligand 1 (PD‐L1) and that the accumulation of PD‐L1‐positive senescent cells increases with the degree of aging.^[^ ^19^ ^]^ As a T cell immune checkpoint molecule, PD‐L1 deactivates tumor‐infiltrating lymphocytes (TILs) that express Programmed cell death 1 (PD‐1) on the cell surface. The interaction between PD‐L1 and PD‐1 inhibits T cell immune activation and effector responses.^[^ ^20^, ^21^ ^]^ Disruption of the PD‐1/PD‐L1 axis and reactivation of TILs are considered promising targets for cancer immunotherapy.^[^ ^22^ ^]^ Since PD‐L1‐negative cells are sensitive to T cell surveillance, PD‐L1‐positive cells are resistant even in the presence of the SASP. Additionally, studies have shown that although cellular senescence is a stable state of cell cycle arrest, this phenomenon is not entirely irreversible, especially in therapy‐induced senescent cells, some of which can escape the state of cell cycle arrest, leading to cancer recurrence.^[^ ^23^ ^]^ Thus, in the context of cancer therapeutics, the discovery of drugs capable of inducing senescence in tumor cells while simultaneously activating the immune system through the downregulation of PD‐L1 expression could significantly enhance the immune response against cancer cells and facilitate the clearance of senescent cells.^[^ ^24^ ^]^The realization of such a strategy would introduce a novel and more potent approach to cancer treatment.

To date, a variety of prosenescence drugs, such as GRN163L, nutlin, and MIRA‐1, have been developed, some of which have entered clinical trials. Nevertheless, their systemic toxicity and drug resistance greatly limit their clinical application.^[^ ^25^, ^26^ ^]^ Although some reports indicate that natural products can decrease the expression of PD‐L1 in tumor cells, their clinical application is unknown.^[^ ^27^, ^28^ ^]^ Drugs that can both induce cellular senescence and reduce the expression of PD‐L1 in tumor cells have rarely been reported. B68 is a compound modified from quaternary isoquinoline alkaloids extracted from natural plants of the Berberidaceae, Ranunculaceae, Rutaceae, and other families. Berberine has been used for the treatment of diseases such as diabetes, gastroenteritis, polycystic ovary syndrome, etc.^[^ ^29^ ^]^ Previous studies have shown that berberine can inhibit the deubiquitination activity of CSN5, reduce the expression of PD‐L1 in cancer cells, and promote antitumor immunity.^[^ ^30^ ^]^ In this study, we found that B68, a derivative modified from berberine, targets CSN5 with an effect similar to that of berberine and can reduce the level of PD‐L1. In addition, B68 induces senescence in cancer cells by targeting BMI1, which berberine cannot do. Our study identified a drug that can promote the senescence of tumor cells while simultaneously decreasing PD‐L1 expression in senescent cells, which will provide new prospects for the treatment of refractory cancer cells.

## 2 Results

### 2.1 B68 Notably Promoted Cell Senescence in Colorectal Cancer Cells

**Figure 1:**
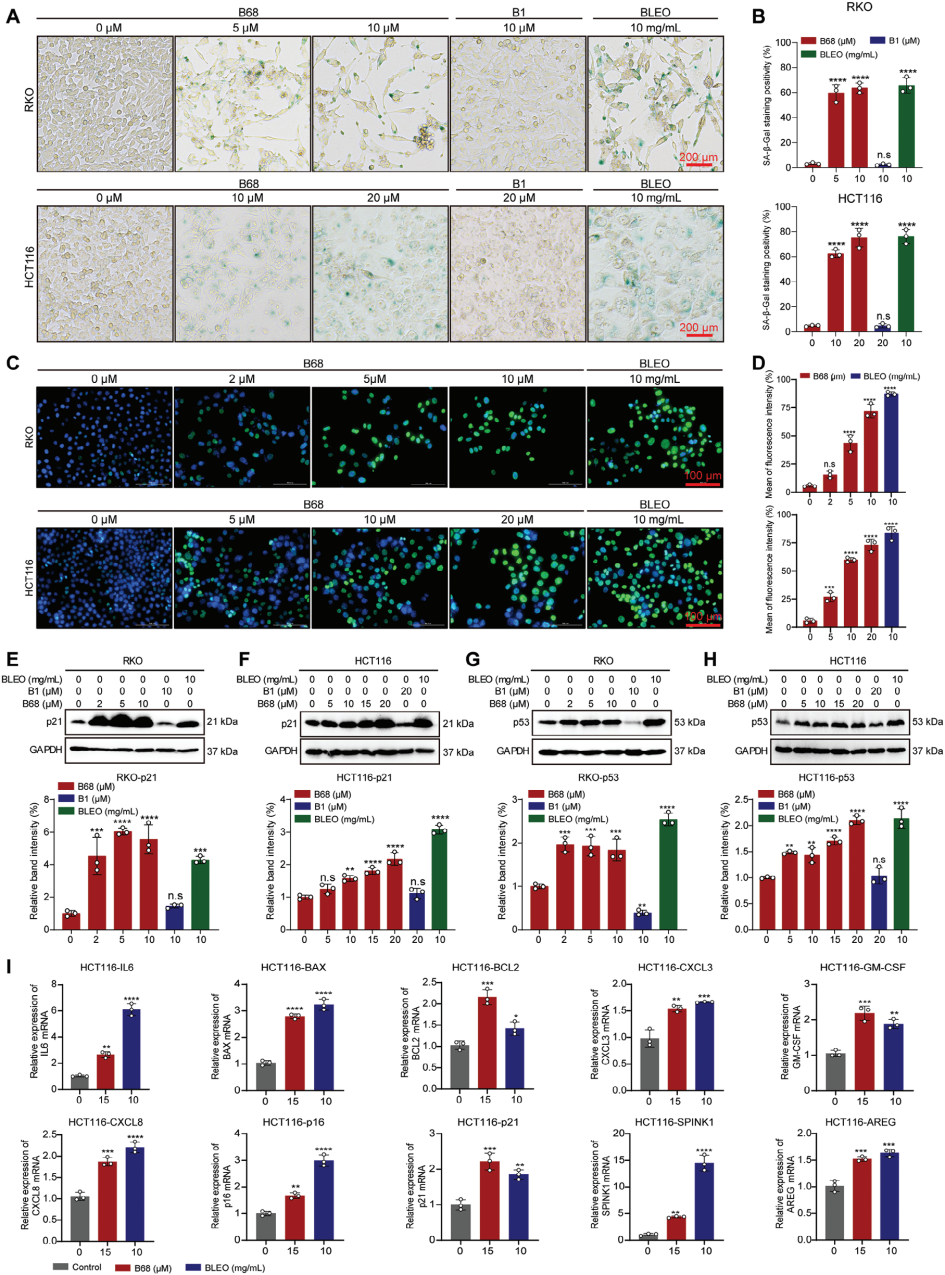
B68 induces senescence in colon cancer cells. A) RKO and HCT116 cells were cultured with different concentrations of B68, B1, and bleomycin (BLEO) for one week, and cellular senescence was assessed by SA‐β‐gal activity staining, and quantification of SA‐β‐gal activity staining is shown in B). Scale bars, 200 µm (*n* = 3, error bars represent SEM, mean ± SEM, one‐way ANOVA). C) Representative images of H3K9me3 staining of RKO and HCT116 cells after 7 days of treatment with B68. Scale bars, 100 µm. D) Quantitative results of H3K9me3 staining in RKO and HCT116 cells (*n* = 3, error bars represent SEM, mean ± SEM, one‐way ANOVA). E–H) The protein expression levels of p21 and p53 were detected by immunoblotting after the treatment of RKO and HCT116 cells with different concentrations of B68 for 7 days. The results of the quantification of the p21 and p53 protein levels are shown below (*n* = 3, error bars represent SEM, mean ± SEM, one‐way ANOVA). I) Quantitative RT‐PCR was conducted to assess the expression levels of mRNAs associated with the SASP following a 7‐day treatment of HCT116 cells with B68 at a concentration of 15 µm and bleomycin at 10 mg mL^−1^ (*n* = 3, error bars represent SEM, mean ± SEM, one‐way ANOVA). n.s, not significant; *p < 0.05, **p < 0.01, ***p < 0.001, ****p < 0.0001.

Considering the limitations of berberine in its antitumor effects, we performed a series of structural modifications to berberine and obtained six products, namely, berberine (B1), B2, B3, B68, B5, B6, and B7.^[^ ^31^ ^]^ Through the CCK‐8 assay, we found that B68 had a significant inhibitory effect on tumor growth in RKO cells compared with that of berberine (B1), with an IC_50_ of 1.03 µm (Figure S1A,B, Supporting Information). Moreover, RKO and HCT116 cells are more sensitive to B68 than other CRC cells (Figure S1C–E, Supporting Information), and B68 (0–20 µm) has almost no toxic effect on normal human intestinal epithelial cells (NCM460) (Figure S1F, Supporting Information). To further assess the growth inhibition effect of B68 on HCT116 and RKO cells, a colony formation assay was performed. The results showed that B68 treatment significantly suppressed the colony‐forming ability of RKO and HCT116 cells (Figure S1G,H, Supporting Information). Next, an EdU assay and calcein‐AM/PI double‐staining assay were performed to analyze the effect of B68 on CRC cell proliferation or cell death. As shown in Figures S1I,J and S2A–D (Supporting Information), B68 significantly inhibited the proliferation of RKO and HCT116 cells with little induction of apoptosis, which is consistent with the flow cytometry results. Moreover, B68 notably increased the percentage of RKO and HCT116 cells in G0/G1 phase, from 33.4% ± 1.3% in controls to 80.9% ± 6.4% and 37.8% ± 2.3% to 60% ± 1.2%, respectively (Figure S2E,F, Supporting Information). Furthermore, we utilized scratch assays to determine the migratory potential of LOVO and CT26 cells. As shown in Figure S3A,B (Supporting Information), the application of B68 resulted in a pronounced reduction in cell migration. Additionally, in RKO and HCT116 cells, B68 significantly altered the cell morphology from spindle‐shaped and round to flat and enlarged, and vacuoles were observed, especially in HCT116 cells, suggesting that B68 might induce cellular senescence (Figure S2G, Supporting Information). Subsequent SA‐β‐gal activity assays and H3K9me3 immunofluorescence experiments also confirmed that B68 significantly promoted RKO and HCT116 cell senescence (**Figure** **1**A–D). It is well documented that cell cycle arrest in senescent cells is predominantly mediated through activation of the p53/p21 signaling pathway. Therefore, to further determine the mechanism of B68‐induced senescence, we evaluated p53 and its downstream target p21. As shown in Figure 1E–H, B68 robustly upregulated the transcription of p53 in CRC cells, consequently promoting the expression of p21. These findings suggest that B68 may initiate a senescence program in CRC cells via activation of the p53/p21 pathway. A prominent feature of cellular senescence is the acquisition of the SASP. We further explored whether B68 affects cellular senescence and SASP regulatory factors. As shown in Figure 1I and Figure S4 (Supporting Information), similar to bleomycin, a positive control drug, B68 significantly increased the levels of IL‐6, BAX, BCL2, CXCL3, GM‐CSF, CXCL8, p16, p21, spink1, and AREG. Taken together, these results indicate that, unlike berberine, the modified berberine derivative B68 can inhibit cell growth by inducing senescence in tumor cells.

### 2.2 B68 Induces Senescence to Suppress Colorectal Cancer Growth in Xenograft Mice

To further determine the antitumor effect of B68‐induced senescence, HCT116 and RKO xenograft nude mouse models were established. Before the formal experiment, our preliminary experiments indicated that 8 mg kg^−1^ B68 had significant antitumor effects (Figure S5A–C, Supporting Information). Therefore, 8 mg kg^−1^ B68 was used in subsequent animal experiments. Notably, B68 markedly suppressed HCT116 and RKO tumor growth, tumor size and tumor weight, whereas berberine had no therapeutic effect (Figures S6A–D and S7A–D, Supporting Information). In addition, after intraperitoneal injection of B68 for 14 days, no significant alterations in average body weight were observed among the treatment groups (Figures S6E and S7E, Supporting Information). B68 treatment significantly increased the percentage of β‐gal‐positive senescent cells in tumor tissue, whereas the control and berberine treatments did not (Figures S6F and S7F, Supporting Information). To evaluate whether the antitumor effect of B68 is attributable to senescence induction, cellular senescence and the expression of the DNA damage sensors γ‐H2AX, p16, p53, and p21 were detected in tumor tissues. As shown in Figures S6G,H and S7G,H (Supporting Information), the expression of γ‐H2AX, p16, p53, and p21 was also markedly increased in the B68 treatment group in both the HCT116 and RKO xenograft mouse models. Furthermore, immunohistochemistry (IHC) showed that the level of Ki‐67 (a marker of proliferation) was decreased, while the level of cleaved caspase‐3 (a marker of apoptosis) was increased in B68‐treated tumor xenograft mice. Moreover, compared with the control treatment, B68 did not markedly alter the pathological changes in major organs, including heart, liver, spleen, lung, and kidney (Figures S6I and S7I, Supporting Information). Collectively, these findings indicate that B68 can suppress the growth of colorectal cancer cells by inducing senescence in tumor cells.

### 2.3 B68 Suppresses Tumor Growth in AOM/DSS‐Induced Colon Cancer

**Figure 2:**
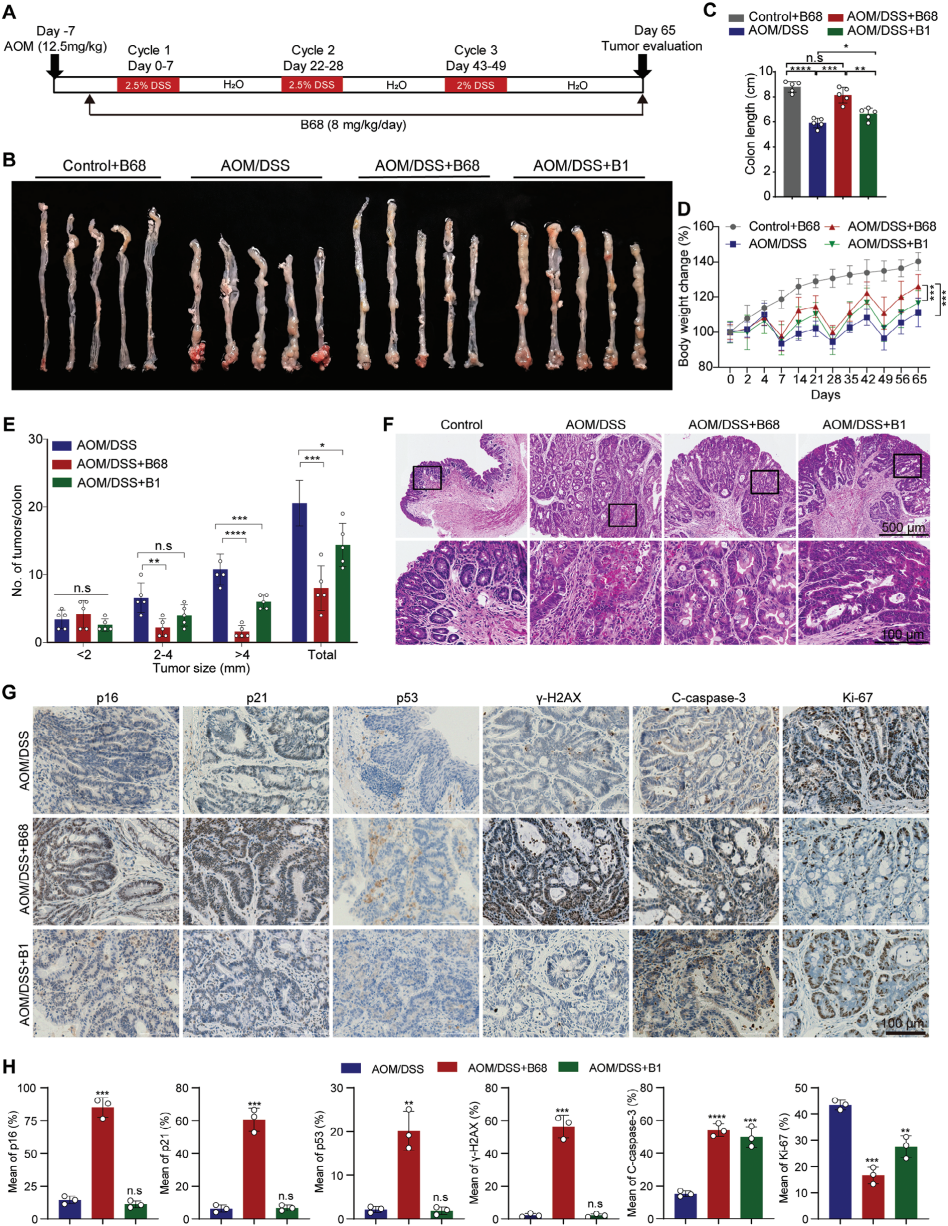
B68 significantly inhibited the development of colorectal cancer induced by AOM/DSS. A) Azoxymethane (AOM‐12.5 mg kg^−1^) was first injected into C57BL/6J (male) mice, followed by 2% or 2.5% glucose sodium sulfate (DSS) in the drinking water for 1 week and then regular drinking water for 2 weeks; this process was repeated three times as described in the “Animal experiment methods” section. B) Representative images of the colon and rectum after AOM/DSS treatment depicting the extent of the tumor load. C) The length of mouse colon in the control group, model group, AOM/DSS+B68 group and AOM/DSS+B1 group (*n* = 5, error bars represent SEM, mean ± SEM, Student's *t*‐test). D) Changes in the body weights of mice in the control, model, AOM/DSS+B68 and AOM/DSS+B1 groups during the experimental period (*n* = 5, error bars represent SEM, mean ± SEM, Student's *t*‐test). E) The number and diameter of tumors in the control group, model group, AOM/DSS+B68 group, and AOM/DSS+B1 group were measured at the end of the treatment (*n* = 5, error bars represent SEM, mean ± SEM, Student's *t*‐test). F) Representative micrographs of H&E staining of colon tumors in control, model, AOM/DSS+B68 and AOM/DSS+B1 mice after AOM/DSS treatment (scale bar, 100 µm or 500 µm). G) Representative images of IHC staining of cleaved caspase 3, Ki‐67, p16, p21, p53, and γ‐H2AX in the colon of model, AOM/DSS+B68 and AOM/DSS+B1 mice; scale bar, 100 µm. H) Quantitative statistical results of the immunohistochemical staining shown in Figure 2G (error bars represent SEM, mean ± SEM, Student's *t*‐test). n.s, not significant; *p < 0.05, **p < 0.01, ***p < 0.001, ****p < 0.0001.

The AOM/DSS model is currently the most widely used chemically induced model for ulcerative colitis (UC). Clinical symptomatology, morphological, and pathological observations have proven that this model is highly similar to the process of cancer in human ulcerative colitis and can be effectively used for the evaluation of antitumor drug efficacy, allowing for the observation of its biological effects in a short period of time. To determine whether B68 can attenuate the formation of CRC, we established an AOM/DSS model and treated the mice with 8 mg kg^−1^ B68 daily via intraperitoneal injection. The specific experimental procedure is shown in **Figure** **2**A. Mice in the AOM/DSS group had a higher tumor burden in the colonic tissue, whereas B68 treatment significantly inhibited the tumors induced by AOM/DSS. Subsequently, our research revealed that B1 is capable of suppressing the development of colorectal cancer, albeit to an extent that is intermediate between the control group and the B68 treatment group, and notably less pronounced than the effects observed in the B68 group (Figure 2B). Quantitatively, treatment with B68 reduced the percentages of large (>4 mm in diameter), medium (2–4 mm in diameter) by 85.2% and 66.7%, respectively, whereas treatment with B1 led to a reduction in the percentages of large (>4 mm in diameter), medium (2–4 mm in diameter), and small (<2 mm) tumors by 44.4%, 39.4%, and 23.5%, respectively, which indicates that the therapeutic efficacy of B68 is significantly superior to that of B1 (Figure 2E). Interestingly, the B68 mice presented a longer colon length and a heavier body weight than the AOM/DSS mice (Figure 2C,D), suggesting that AOM/DSS mice are more likely to develop tumors, as DSS‐induced shortening of colon length in mice is an indicator of the severity of inflammation, a potential cause of colon carcinogenesis. Furthermore, H&E findings indicated that treatment with B68 in mice also substantiated the effectiveness of B68 therapy, indicating that the therapeutic effects of B68 is superior to B1 (Figure 2F). Moreover, IHC also showed that B68 could increase the expression of the protein senescence‐related markers γ‐H2AX, p16, p53, and p21. Concurrently, the expression of Ki‐67, which is associated with proliferation, decreased, whereas the expression of cleaved caspase‐3, which is related to apoptosis, significantly increased (Figure 2G,H). Interestingly, following treatment with B68, there was a significant increase in the number of CD8^+^ cells within the tumor, while the levels of PD‐L1 and Foxp3 were significantly reduced. The levels in the group treated with B1 were intermediate between those in the control and B68 groups (Figure S8A,B, Supporting Information). Additionally, B68 did not significantly change the tissue indices of the heart, liver, spleen, lungs, or kidneys compared with those in the control treatment group (Figure S8C, Supporting Information). Taken together, these results suggest that B68 suppresses AOM/DSS‐induced carcinogenesis by promoting tumor cell senescence.

### 2.4 B68 Induces Senescence in Colorectal Cancer Cells by Targeting BMI1

**Figure 3:**
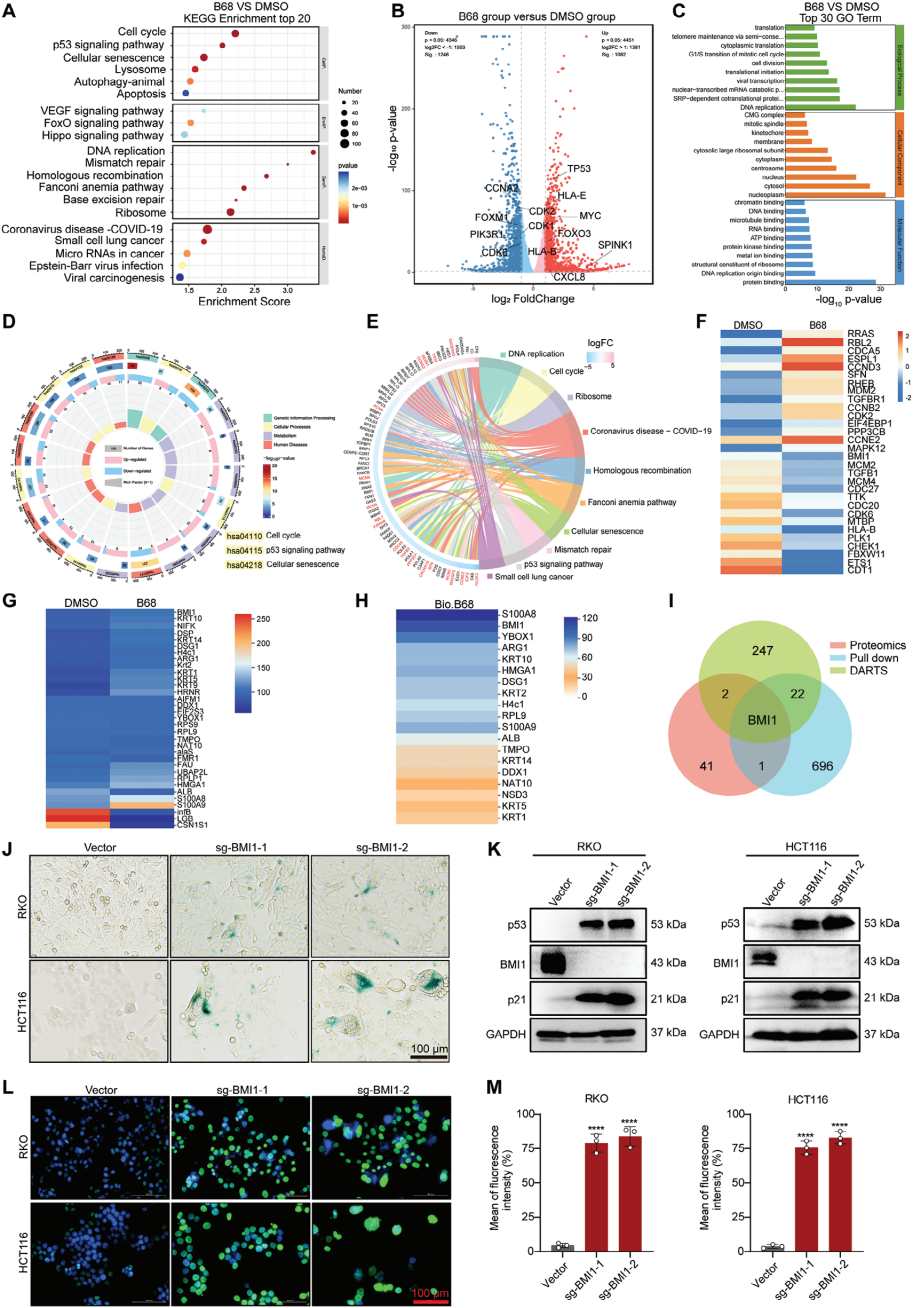
B68 exerts antitumor effects by targeting BMI1‐induced senescence in colorectal cancer cells. A) KEGG analysis of differentially expressed mRNAs in B68‐treated RKO cells. B) The volcano plot illustrates the changes in mRNA levels after B68 treatment in RKO cells (red dots represent significant upregulation, and blue dots represent significant downregulation), with marked genes related to senescence and cell cycle (fold change ≥2, p value ≤0.05). C) Gene Ontology (GO) analysis of differentially expressed mRNAs in RKO cells after B68 treatment. D,E) KEGG enrichment of the potentially differentially expressed proteins associated with senescence and cell cycle in B68‐treated RKO cells. F) Heatmap showing the expression of the top 30 differentially expressed proteins in B68‐treated RKO cells, with senescence‐ and cell cycle‐related genes labeled (fold change ≥1.2, unique peptides ≥2). G) DARTS analysis of potential target proteins that bind to B68. H) Pull‐down analysis of potential target proteins that bind to B68. I) Venn analysis results of differentially expressed proteins that appear in the DARTS, pull‐down and proteomics results. Detection of SA‐β‐gal activity J), p21 and p53 protein expression K) following BMI1 gene knockdown in RKO and HCT116 cells. L) H3K9me3 expression in RKO and HCT116 cells after BMI1 gene knockdown was detected by immunofluorescence, and the results of the quantitative analysis are shown on the right (M). Scale bars, 100 µm (*n* = 3, error bars represent SEM, mean ± SEM, one‐way ANOVA). ****p < 0.0001.

To explore the molecular mechanism of B68‐induced cell senescence, we performed transcriptome sequencing of B68‐treated RKO cells. By KEGG pathway enrichment analysis (**Figure** **3**A), the top three enriched pathways were identified as the cell cycle, p53 signaling pathway, and cellular senescence pathway, which was consistent with the senescence phenotype induced by B68 and further confirmed that B68 triggers cellular senescence. In the B68‐treated group, the mRNA levels of 1050 genes increased, and the mRNA levels of 1221 genes decreased. Among these genes, several senescence‐associated genes, including CXCL8, SPINK1, TP53, MYC, and FOXO3, were upregulated, whereas others, including CCNA2, FOXM1, PIK3R1, and CDK8, were downregulated (Figure 3B). Additionally, GO analysis confirmed the activation of the cellular senescence pathway (Figure 3C). Considering that B68‐induced cellular senescence leads to changes in the levels of senescence‐associated proteins, we also conducted a proteomic analysis of B68‐treated cells. As anticipated, the cell cycle, p53 signaling pathway, and cellular senescence pathway were the most enriched pathways (Figure 3D,E). We subsequently focused on genes responsible for the activation of senescence‐associated proteins and observed significant upregulation or downregulation of a series of senescence‐associated proteins, such as CCND3, RBL2, CCNE2, and CDK2 (Figure 3F). In conclusion, these results further indicate that B68 mediates the death of tumor cells through senescence.

**Figure 4:**
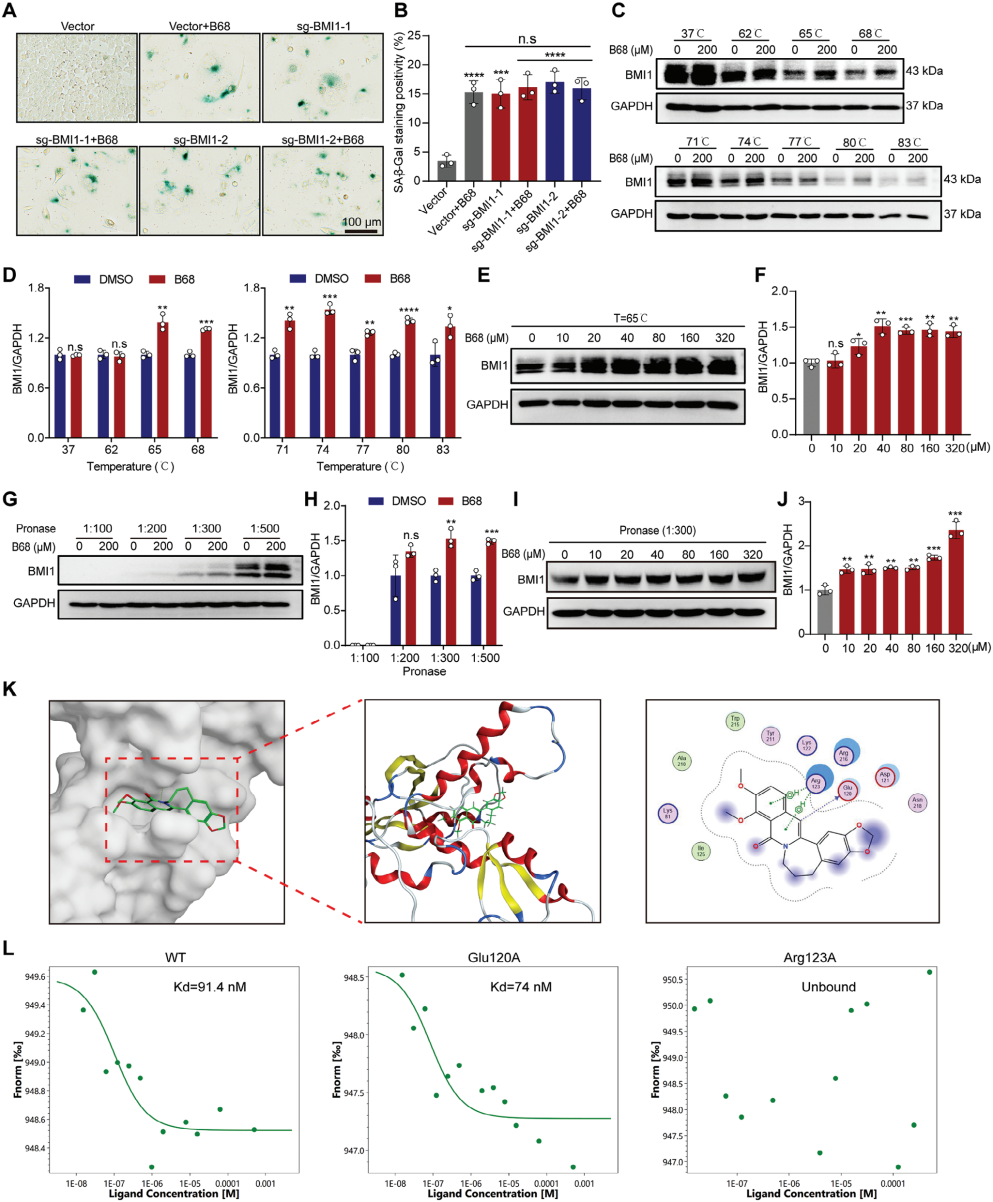
B68 induces senescence in colorectal cancer cells by targeting BMI1. A) After BMI1 knockdown, B68 did not further increase SA‐β‐Gal activity in HCT116 cells. B) Quantitative results of SA‐β‐Gal staining are shown on the right (*n* = 3, error bars represent SEM, mean ± SEM, one‐way ANOVA). C) CETSA was used to determine the thermal stabilization of the BMI1 interaction with B68 at a series of temperatures from 62 to 83 °C, and the quantification of BMI1 is shown in (D) (*n* = 3, error bars represent SEM, mean ± SEM, Student's *t*‐test). E) Stability of different concentrations of B68 on BMI1 at 65 °C. F) Quantitative results of BMI1 intensity in (E) (*n* = 3, error bars represent SEM, mean ± SEM, Student's *t*‐test). G) Effect of B68 on the stability of the BMI1 protein at different protein hydrolase concentrations. H) Quantitative results of BMI1 intensity in (F) (*n* = 3, error bars represent SEM, mean ± SEM, Student's *t*‐test). I) Effect of different concentrations of B68 on BMI1 protein stability at a pronase‐to‐protein ratio of 1:300. The quantitative results for the BMI1 protein are shown on the right (J) (*n* = 3, error bars represent SEM, mean ± SEM, Student's *t*‐test). (K) Molecular docking of B68 with BMI1. L) Cellular MST assay of GFP‐tagged BMI1 upon overexpression of wild‐type BMI1 and disrupted mutants (Arg123 or Glu120). n.s, not significant; *p < 0.05, **p < 0.01, ***p < 0.001, ****p < 0.0001.

To explore the targets of B68‐induced tumor cell senescence, we utilized drug affinity responsive target stability (DARTS) to identify protein targets that directly interact with B68. The workflow of DARTS is shown in Figure S9A (Supporting Information). DARTS predicted 33 potential target proteins (unique peptide 2, with an intensity ratio of 1.5) (Figure 3G). We subsequently used B68 as a chemical probe to explore potential targets involved in the senescence of RKO cells. First, we synthesized biotin‐B68, which results in a senescent phenotype in RKO cells. An in vitro pull‐down assay was conducted using Bio‐B68 (or Bio as a negative control) with cancer cell lysates (Figure S9B, Supporting Information). However, in the SDS‐PAGE analysis, no differential bands were detected by silver staining (Figure S9C Supporting Information,). Notably, the absence of specific bands does not indicate a lack of target pulling; hence, we subjected the entire lane to mass spectrometry analysis. The results revealed 15 potential target genes of B68 through pull‐down analysis (Figure 3H). By analyzing the results of the DARTS and pull‐down experiments, we identified four aging‐related genes: UBAP2L, YBOX1, BMI1, and S100A9. Subsequently, we conducted interference experiments on these four genes and discovered that the senescent phenotype was observed exclusively upon BMI1, which is consistent with the intersecting results from DARTS, proteomics and pull‐down assays related to senescence (Figure 3I; Figure S9D–H, Supporting Information). Furthermore, changes in the expression of BMI1 in RKO and HCT116 cells were detected by immunoblotting (IB). Treatment with B68 significantly reduced BMI1 expression in both cell lines in a time‐ and concentration‐dependent manner. Additionally, B68 had no effect on the BMI1 mRNA level, suggesting that B68 induces senescence in tumor cells by degrading BMI1 (Figure S10, Supporting Information). Next, we knocked out BMI1 in both RKO and HCT116 cells. After gene knockout, both cell lines exhibited a pronounced senescence phenotype, with significant upregulation of SA‐β‐gal activity or p53 or p21 (Figure 3J,K; Figure S9I–L, Supporting Information). Consistent results were also obtained from H3K9me3 immunofluorescence assays, which demonstrated that BMI1 knockout significantly promoted the senescence of RKO and HCT116 cells (Figure 3L,M). Additionally, after the knockout of BMI1 followed by treatment with B68, B68 did not further promote cellular senescence in HCT116 cells (**Figure** **4**A,B).

### 2.5 ARG123 of BMI1 is Critical for Binding to B68

To ascertain whether B68 binds directly to the BMI1 protein, we employed cellular thermal shift assay (CETSA) technology, a technique grounded in the principle that the thermal stability of the target protein is enhanced upon ligand binding. Given that prolonged protein‐drug incubation in lysates could lead to protein‐metabolite interactions, we opted for a 1‐minute incubation to detect direct binding. As depicted in Figure 4C,D, B68 notably increased BMI1 accumulation across temperatures ranging from 37 to 83 °C, implying that B68 directly interacts with BMI1 by modulating its thermal stability. To corroborate the CETSA findings, we subjected the lysate to B68 at varying concentrations at 65 °C and observed increased BMI1 stability with increasing B68 levels (Figure 4E,F). In line with the CETSA results, BMI1 accumulation increased with increasing B68 concentration at an enzyme‐lysate ratio of 1:300 (Figure 4G–J). To determine the BMI1 amino acid residues involved in B68 binding, we conducted a docking simulation via Maestro software (Schrödinger, version 9.0) to model the binding mode of B68 to BMI1 (Figure 4K). Subsequently, we performed microscale thermophoresis (MST) to analyze the direct binding between B68 and exogenous, GFP‐tagged BMI1 protein. As anticipated, B68 strongly bound to BMI1, with a Kd value of 91.4 nm. Figure 4L illustrates the formation of two stable hydrogen bonds between the backbone of BMI1 at positions Glu120 and Arg123 with B68. We then conducted cellular MST assays on overexpressed GFP‐tagged BMI1, including wild‐type and disruptive mutants at positions Glu120 and Arg123. Although the mutation of Glu120 to alanine resulted in a Kd nearly identical to that of the wild type, the mutation of Arg123 to alanine completely abrogated BMI1‐B68 complex formation.

### 2.6 B68 Induces the Immunogenicity of Cellular Senescence and Promotes Antitumor Immunity

**Figure 5:**
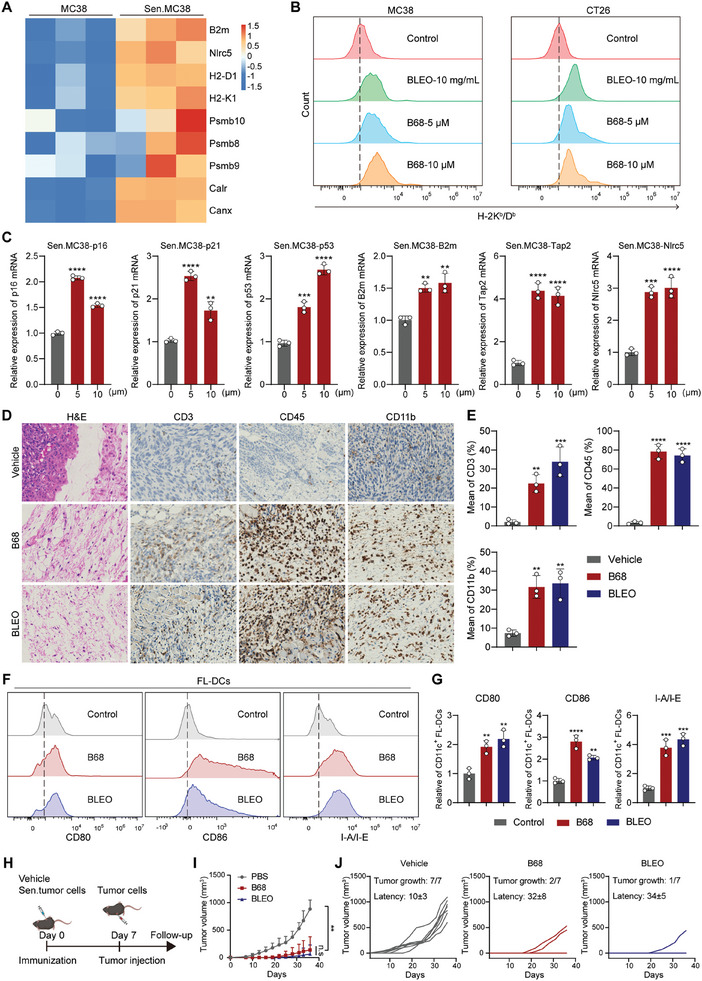
Immunization with senescent tumor cells promotes antitumor effects and immune surveillance. A) RNA‐seq analysis of the mRNA expression of genes related to antigen presentation mechanisms and immunity in MC38 cells in the control and B68 groups. B) Flow cytometry detection of H‐2K^b^/D^b^ expression in B68‐treated MC38 and CT26 cells. C) After senescence was induced in MC38 cells with B68, the mRNA expression of the p16, p21, p53, B2 m, Tap2, and Nlrc5 genes was detected by qRT‐PCR (*n* = 3 independent experiments, error bars represent SEM, mean ± SEM, one‐way ANOVA). D) C57BL/6 mice were subcutaneously injected with either vector (PBS) or senescent MC38 cells. On day 7, the skin at the site of tumor inoculation was subjected to H&E staining and immunohistochemistry, with a scale = 100 µm. The quantitative results of immunohistochemistry are shown on the right E) (*n* = 3, error bars represent SEM, mean ± SEM, one‐way ANOVA). F) Flow cytometry analysis of CD80, CD86, and MHC‐II (I‐A/I‐E) expression in DCs cocultured with senescent MC38 cells and quantification of CD80, CD86, and MHC‐II (I‐A/I‐E) expression are shown on the right G) (*n* = 3, error bars represent SEM, mean ± SEM, one‐way ANOVA). H) C57BL/6J mice were subcutaneously injected with PBS or senescent MC38 cells on the left side, and on day 7, MC38 cells were injected on the right side, after which the growth of tumors on the right side of the mice was observed. I,J) Individual tumor growth curves of PBS‐treated mice or mice immunized with senMC38 cells. Tumor growth (number of animals that developed tumors out of the total) and tumor latency (mean ± SEM of the day on which the tumor appeared) are indicated for each group. One‐way ANOVA was performed, and the error bars represent SEM, mean ± SEM). n.s, not significant; **p < 0.01, ***p < 0.001, ****p < 0.0001.

Previous studies have shown that senescent cancer cells can elicit antitumor protection when used in a vaccination setting, a characteristic attributed to their SASP. Next, we initially assessed the expression of SASP in response to B68 in MC38 cells. As the transcriptomic results showed, compared to those in nonsenescent controls, an upregulation of the interferon (IFN) transcriptional signature, including the major gene components involved in MHC‐I antigen processing and presentation, was observed in senescent cells (**Figure** **5**A). The upregulation of the majority of senescence‐associated genes, particularly the master transcriptional regulators of MHC‐I‐dependent immune responses B2 m, Nlrc5, and Tap2, was confirmed through qRT‐PCR analysis of separate biological replicates (Figure 5C). Senescent cells upregulate MHC‐I antigen presentation, and to determine whether the transcriptional upregulation of MHC1‐related genes is reflected in an increase in antigen presentation, we examined the expression of H‐2K^b^/D^b^ in CT26 and MC38 cells and HLA‐A/B/C in RKO and HCT116 cells via flow cytometry. The results showed that the expression of H‐2K^b^/D^b^ and HLA‐A/B/C significantly increased in senescent cells treated with B68, suggesting that B68 enhances the ability of senescent cells to process and present MHC‐I antigens (Figure 5B; Figure S11A–C, Supporting Information). After demonstrating the enhanced immunogenicity of senescent cells, we turned our attention to cancer research with the ultimate goal of using senescent cancer cells to elicit protective antitumor immune responses. To determine whether the secretions from senescent cells reflect an enhancement in immune recruitment in vivo, we injected mice subcutaneously with either vehicle (PBS) or senescent MC38 cells and performed histological analysis on the injection sites. Interestingly, one week post‐injection, the subcutaneously injected senescent MC38 cells were clearly visible as large pleomorphic and anaplastic cells surrounded by a rich influx of immune cells (CD45^+^), primarily myeloid (CD11b^+^) and T cells (CD3^+^) (Figure 5D,E). Following the recruitment of immune cells, the next crucial step in triggering an adaptive immune response includes the capture of antigens by dendritic cells (DCs), as well as the activation and maturation of DCs. To test this possibility, we cocultured dendritic cells with untreated or senescent MC38 cells and examined the surface expression of the DC activation and maturation markers CD80, CD86, and MHC‐II (I‐A/I‐E) on the DCs. The cells induced to senesce with bleomycin served as a positive control for DC activation. We found that coculturing dendritic cells with senescent MC38 cells, as well as B68, both increased the expression of DC maturation markers CD80, CD86, and I‐A/I‐E (Figure 5F,G). Given that senescent cells exhibit increased antigenicity and adjuvanticity, we hypothesize that senescent cancer cells could be utilized to promote an immune response against cancer. Here, we initially immunized B68‐induced senescent MC38 cells to assess the preventive effects against subsequent proliferation upon rechallenge with MC38 cells. One week after subcutaneous immunization, all the experimental groups received a subcutaneous injection of 1.5 × 10^5^ untreated MC38 cells on the contralateral side (Figure 5H). We observed that immunization with sen‐MC38 provided robust antitumor protection (Figure 5I,J). Taken together, our observations suggest that immunization with B68‐induced senescent cancer cells can promote an antitumor immune response that contributes to tumor prevention.

### 2.7 B68 Mediates PD‐L1 Degradation by Targeting CSN5

**Figure 6:**
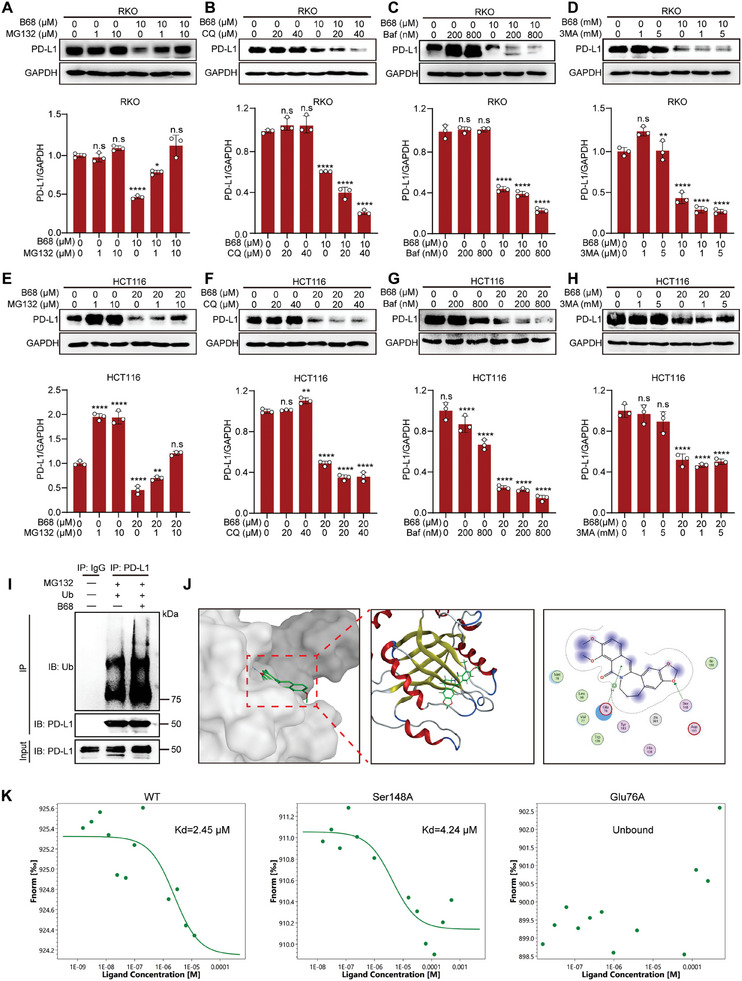
B68 targets CSN5 to decrease the expression of PD‐L1 on tumor cells. A–H) Immunoblotting for PD‐L1 expression in RKO and HCT116 cells treated with B68 in combination with the proteasome inhibitor MG132 (1 µm, 10 µm), chloroquine (20 µm, 40 µm), the lysosomal inhibitor bafilomycin A1 (200 nm, 800 nm) or the autophagy inhibitor 3‐methyladenine (1 mm, 5 mm). The quantitative results of PD‐L1 expression are shown in the figure below (*n* = 3, error bars represent SEM, mean ± SEM, one‐way ANOVA). I) Immunoprecipitation (IP) analysis of PD‐L1 ubiquitination in RKO cells treated with B68 and IB analysis with a ubiquitin antibody. The cells were treated with MG132 before ubiquitination analysis was performed. J) Molecular docking of B68 with CSN5. K) The binding of CSN5 to B68 was determined by MST after the overexpression of GFP‐tagged wild‐type CSN5 or disruptive mutants (Ser481 and Glu76). n.s, not significant; *p < 0.05, **p < 0.01, ****p < 0.0001.

Considering that berberine can modulate the expression of PD‐L1 to exert an antitumor immune effect, we also investigated whether compound B68 can reduce PD‐L1 levels. As shown in Figure S12A–L (Supporting Information), the WB, flow cytometry, and immunofluorescence results all showed that B68 dose‐dependently reduced the expression of PD‐L1 in RKO and HCT116 cells, indicating that B68 also has an antitumor immune effect by reducing PD‐L1. Subsequently, we tested whether B68 could diminish the ability of cancer cells to bind to PD‐1. After incubating B68‐treated RKO cells with recombinant PD‐1 protein and fluorescent antibodies, we used confocal microscopy to detect the interaction between PD‐L1 and PD‐1. The reduced green fluorescence in B68‐treated RKO cells indicated that B68, by lowering PD‐L1 levels, diminished the binding capacity of cancer cells to PD‐1 (Figure S13A,B, Supporting Information). Similarly, coculture experiments of B68‐treated RKO and HCT116 cells with T cells demonstrated that B68 can reduce the viability of RKO and HCT116 cells in a dose‐dependent manner, indicating that B68 can exert an antitumor immune effect by reducing the expression of PD‐L1 (Figure S13C–F, Supporting Information). To further explore the mechanism by which B68 affects the expression of PD‐L1, we measured the expression levels of PD‐L1 mRNA in colorectal cancer cells after treatment with B68 or treated tumor cells in combination with the protein synthesis inhibitor cycloheximide (CHX) to assess the changes in PD‐L1 protein expression. These findings indicate that, similar to berberine, B68 does not directly affect the transcriptional level of PD‐L1 but modulates its expression through posttranslational modifications (Figure S14A–H, Supporting Information). Regardless of whether B68, like berberine, mediates the degradation of PD‐L1 via the ubiquitin‒proteasome pathway, the results showed that the accelerated degradation of PD‐L1 in RKO cells induced by B68 can be reversed by MG132, while CQ, 3MA and Baf cannot, and the increased ubiquitination of PD‐L1 by B68 confirmed this point (**Figure** **6**A–I; Figure S14I,J, Supporting Information). Finally, molecular docking and microscale thermophoresis (MST) experiments revealed that B68 may mediate the degradation of PD‐L1 by targeting the Glu76 site of the CSN5 protein (Figure 6J–K). Additionally, our immunoprecipitation (IP) experiments confirmed the interaction between CSN5 and PD‐L1 (Figure S14K,L, Supporting Information), further confirming that B68 mediates the ubiquitination‐mediated degradation of PD‐L1 by targeting CSN5.^[^ ^32^ ^]^

### 2.8 B68 Suppresses MC38 Tumor Growth by Promoting Tumor Cell Senescence and Enhancing T Cell Infiltration

**Figure 7:**
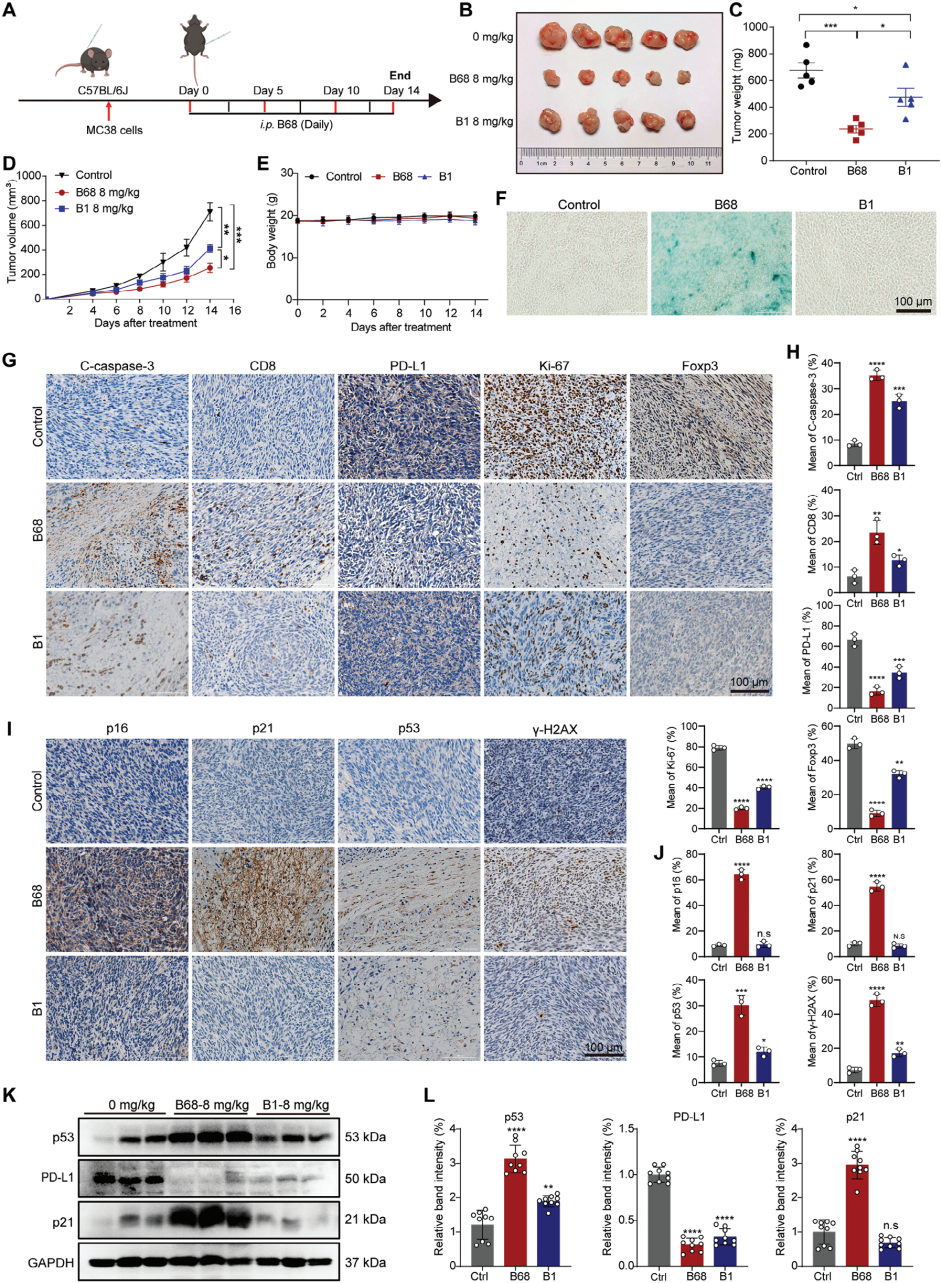
B68 is capable of inducing senescence in colorectal cancer cells and exerting antitumor activity by diminishing PD‐L1 expression on tumor cells. A) MC38 cells were injected into C57BL/6 mice on day ‐5, and B68 (8 mg kg^−1^) and B1 (8 mg kg^−1^) were administered as indicated. B) Ex vivo observation of the tumors. C) Tumor weight, D) tumor volume, and E) weight of the treated mice. F) After the experiment, the tumors were removed, and frozen sections were prepared for SA‐β‐Gal staining, scale bar, 100 µm. G–J) Representative IHC staining results for PD‐L1, CD8, cleaved caspase‐3, Foxp3, Ki‐67, p16, p21, p53, and γ‐H2AX in tumor tissues and quantitative results of immunohistochemical analysis are shown on the right. Scale bar, 100 µm (error bars represent SEM, mean ± SEM, Student's *t*‐test). K) Western blot analysis of p21, p53 and PD‐L1 expression in the tumor tissues of C57BL/6J mice. L) The results of the quantitative analysis are shown in the right (error bars represent SEM, mean ± SEM, one‐way ANOVA). n.s, not significant; *p < 0.05, **p < 0.01, ***p < 0.001, ****p < 0.0001.

Considering that berberine can exert antitumor immune effects by regulating PD‐L1, we then conducted animal experiments on C57BL/6J mice with a normal immune system (**Figure** **7**A). By analyzing tumor volume, weight, and size, we found that both B68 and B1 significantly inhibited the growth of the MC38 xenograft, with B68 showing better therapeutic effects than berberine treatment (Figure 7B–E). To explore whether the superior therapeutic effect of B68 compared to B1 is caused by the induction of tumor cell senescence, we measured indicators related to senescence and PD‐L1 in the tumor tissue, respectively. As shown in Figure 7F–H, compared with the control group, both berberine and B68 significantly reduced the expression of PD‐L1. However, B68 increased the percentage of β‐galactosidase‐positive senescent cells in the tumor tissue, whereas berberine did not, indicating that B68 exerts dual antitumor effects by inducing tumor cell senescence and reducing tumor PD‐L expression. To verify this hypothesis, we subsequently conducted IHC and WB analyses on the tumor tissue and found that both B68 and B1 significantly reduced the expression of PD‐L1 in the tumor tissue, as well as the expression of immune‐suppressive factors such as Foxp3. Moreover, by analyzing the senescence‐associated markers γ‐H2AX, p16, p21, and p53, we found that only B68 increased the protein expression of γ‐H2AX, p16, p21, and p53, which was consistent with the results in nude mice (Figure 7I,J). We also assessed the changes in PD‐L1, p21, p53, and ubiquitinated protein levels in tumor tissues from MC38 subcutaneous tumor‐bearing mice treated with B68 (8 mg kg^−1^) or B1 (8 mg kg^−1^). As shown in Figure 7K,L and Figure S15A (Supporting Information), only B68 was able to increase the protein expression of the senescence markers p21 and p53. Moreover, both B68 and B1 were found to reduce PD‐L1 protein levels by increasing the ubiquitination levels of PD‐L1.

Moreover, the immunohistochemical results also showed no obvious toxicity in the heart, liver, spleen, lung, or kidney (Figure S15B, Supporting Information), indicating that B68 could exert an inhibitory effect on colorectal cancer both through senescence and by reducing the expression of tumor PD‐L1.

### 2.9 B68 Can Enhance the Anti‐CTLA4‐Mediated Inhibition of CRC Effects

**Figure 8:**
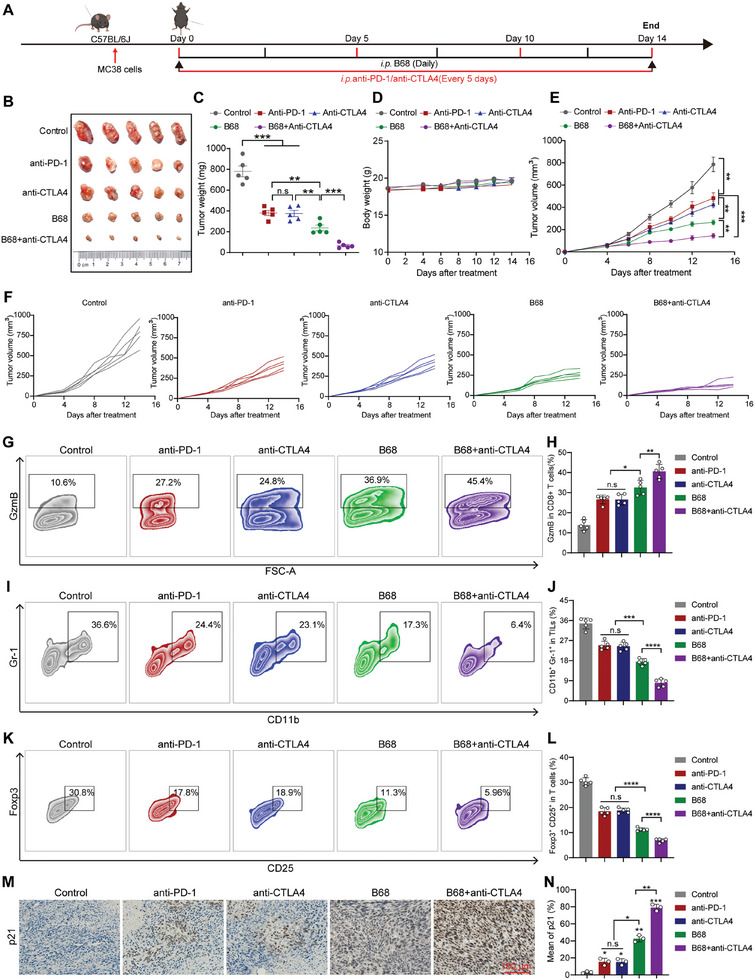
B68 in combination with CTLA4 antibody effectively inhibited tumor growth in vivo in immunoreactive mice. A) C57BL/6 mice harboring MC38 cell‐derived tumors were subjected to various treatments, including PBS, anti‐PD‐L1, anti‐CTLA4, B68 alone, and a combination of B68 with anti‐CTLA4. On day 5, MC38 cells were implanted into C57BL/6 mice, followed by the administration of B68, anti‐PD‐L1, or anti‐CTLA4 at the indicated time points. Subsequently, the tumor morphology B), tumor mass C), body weight D), and tumor volume E) were monitored and measured over a 14‐day period. F) Xenograft tumor growth curves of C57BL/6J mice in each treatment group (*n* = 5, error bars represent SEM, mean ± SEM, Student's *t*‐test). Flow cytometric analysis was utilized to quantify the presence of GzmB^+^ cells F), Gr‐1^+^CD11b^+^ myeloid cells G), and Foxp3^+^CD25^+^ regulatory T cells H) among the groups treated with PBS, anti‐PD‐L1, anti‐CTLA4, B68 alone, B68 combined with anti‐PD‐L1, or B68 combined with anti‐CTLA4, and the quantitative results of the flow cytometry analysis are shown on the right (*n* = 5, error bars represent SEM, mean ± SEM, Student's *t*‐test). M) Representative immunohistochemical images of p21 in the tumor tissue of mice in each group, and the quantitative results of immunohistochemistry are shown on the right. Scale bar, 100 µm (*n* = 3, error bars represent SEM, mean ± SEM, Student's *t*‐test). n.s, not significant; *p < 0.05, **p < 0.01, ***p < 0.001, ****p < 0.0001.

Considering that B68 can induce cellular senescence while reducing the expression of PD‐L1 in tumor cells and that combination therapy with anti‐PD‐L1 and anti‐CTLA4 has better therapeutic effects, we hypothesize that the combination of B68 with anti‐CTLA4 may further enhance B68 antitumor activity. Next, we sought to determine whether B68 could synergize with anti‐CTLA4 to inhibit tumor growth in vivo by using an MC38 mouse cancer model. A schematic representation of the model is depicted in **Figure** **8**A. The results revealed that B68 significantly suppressed tumor growth without affecting body weight, which was better than the effect of anti‐PD‐1 and anti‐CTLA4, suggesting that B68 suppresses tumor growth through senescence in addition to its immune checkpoints (Figure 8B–D). We further found that cotreatment with B68 and anti‐CTLA4 significantly reduced the size of the tumors compared to treatment alone or anti‐CTLA4 monotherapy (Figure 8E,F). Consequently, the combination therapy led to a further increase in granzyme B production within the tumor (Figure 8G,H). Myeloid‐derived suppressor cells (MDSCs) and regulatory T cells (Tregs) are recognized as the primary immunosuppressive elements in the tumor microenvironment and contribute to tumor immune evasion by suppressing T lymphocyte responses. Flow cytometry analysis also revealed that the combination treatment significantly decreased the accumulation of MDSCs, identified as CD11b^+^Gr1^+^ cells, and Tregs, characterized by a CD4^+^CD25^+^ Foxp3^+^ phenotype, compared to the single treatment group (Figure 8I–L). In addition, IHC showed decreased levels of PD‐L1 and ki‐67 (a marker of proliferation) and increased caspase 3 levels in tumor‐xenografted mice, indicating that B68 triggered significant apoptosis in tumor‐bearing mice. Moreover, an analysis of the senescence‐related markers γ‐H2AX, p16, p21, and p53 revealed that, as expected, B68 increased the expression of these proteins, and H&E staining revealed that B68 had no significant toxic effects on C57BL/6J mice (Figure 8M,N; Figure S16A–C, Supporting Information). In brief, these findings collectively demonstrate that B68 inhibits the progression of CRC by inducing cellular senescence and reducing the levels of PD‐L1 in the tumor microenvironment and can exert synergistic antitumor effects when combined with CTLA‐4 antibody therapy, thereby further enhancing the response against cancer.

### 2.10 The Protein Expression Levels of BMI1, CSN5, and PD‐L1 in Human Tumor Tissue

**Figure 9:**
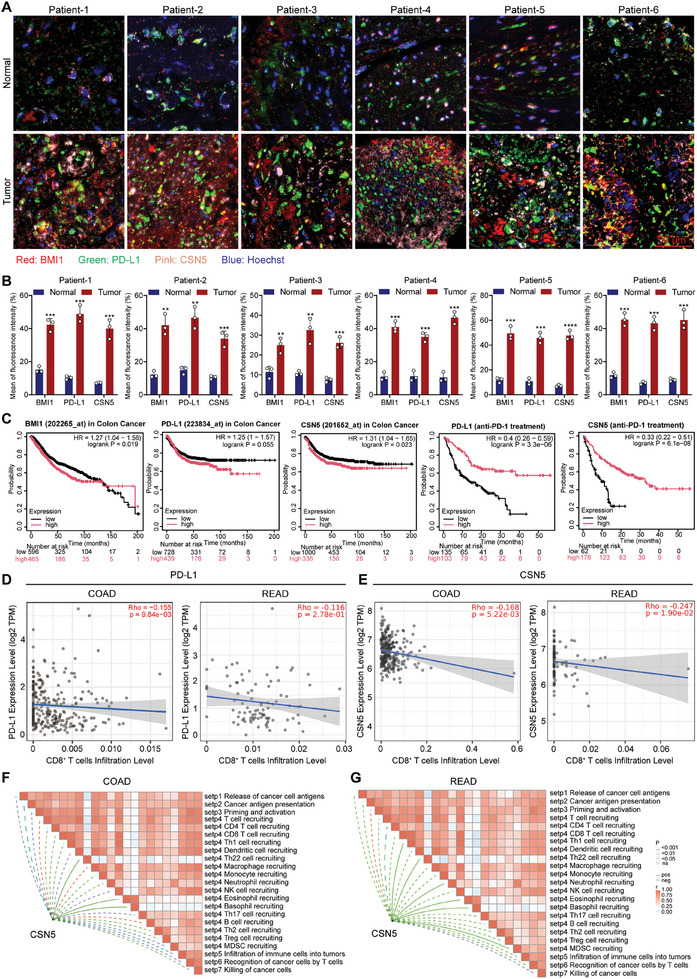
Clinical relevance of BMI1, PD‐L1, or CSN5 in colorectal cancer tissues. A) Representative fluorescence images of BMI1, CSN5, or PD‐L1 in paracancerous tissue (Normal) and tumor tissue (Tumor) of CRC patients. B) Quantification of BMI1, CSN5, or PD‐L1 expression in (A) (*n* = 3, error bars represent SEM, mean ± SEM, Student's *t*‐test). C) The survival of CRC patients stratified by the expression of CSN5 or PD‐L1 was compared by two‐sided log‐rank analysis. D,E) Scatter plot showing the correlation between PD‐L1 or CSN5 expression and infiltrating CD8^+^ T cells in COAD and READ patients detected by TIMER 2.0. F,G) Correlations between CSN5 and the steps of the cancer immune cycle in COAD and READ patients. **p < 0.01, ***p < 0.001, ****p < 0.0001.

To confirm the validity of the aforementioned model in human tumor tissues, we evaluated the protein levels of CSN5, BMI1, and PD‐L1 in both paracancerous and tumor tissues extracted from six colon cancer patients. The findings, as depicted in **Figure** **9**A,B, revealed a marked increase in the expression levels of these proteins in the cancerous tissues compared with the surrounding noncancerous tissues. To delineate the correlation between the expression patterns of CSN5, BMI1, and PD‐L1 in the tumor tissues of colorectal cancer patients and their prognostic implications, we analyzed overall survival data from the TCGA colorectal cancer database. The data analysis indicated that patients exhibiting elevated levels of PD‐L1 and CSN5 had poorer clinical outcomes than did those who received PD‐1 antibody therapy, who exhibited an extended survival period (Figure 9C). Furthermore, leveraging the TIMER 2.0 database, we scrutinized the interplay between the expression of PD‐L1 and CSN5 and the degree of immune cell infiltration within colon (COAD) and rectal adenocarcinomas (READ), respectively. This analysis revealed an inverse relationship between the expression of these proteins and the infiltration of CD8^+^ T cells within the tumor immune microenvironment (Figure 9D,E). Consistent with our hypotheses, CSN5 was inversely correlated with pivotal elements of the cancer‐immunity cycle, including the release of tumor antigens, the priming and activation phase, the trafficking of immune cells to the tumor site, including the recruitment of CD4^+^ T cells, macrophages, dendritic cells, and NK cells, as well as the T cell‐mediated recognition of cancer cells in COAD and READ, with a pronounced negative correlation in COAD (Figure 9F,G). Overall, elevated BMI1 expression was correlated with an adverse prognosis in colorectal cancer patients. The overexpression of CSN5 or PD‐L1 was associated with tumor‐induced immunosuppression, while patients receiving PD‐1 antibody treatment exhibited prolonged survival.

## 3 Discussion

Cellular senescence refers to the attainment of a stable state of cell cycle arrest, which effectively inhibits the further proliferation of damaged or aged cells and plays a critical role in tumor suppression.^[^ ^33^ ^]^ Although cellular senescence is generally considered irreversible, under specific conditions, particularly in tumor cells, reentry into the cell cycle is possible. Consequently, researchers have proposed a strategy that involves inducing senescence in tumor cells followed by the clearance of these senescent cells, which can effectively control the proliferation of premalignant cells and thus prevent the formation and progression of malignant tumors.^[^ ^34^, ^35^, ^36^, ^37^ ^]^ Here, we found that B68, a derivative modified from berberine, can induce senescence in colorectal cancer cells by targeting BMI1, exerting an antitumor effect. Moreover, B68 can reduce the expression of PD‐L1 on the surface of senescent cells by targeting CSN5, thereby blocking the direct interaction between PD‐1 on T cells and PD‐L1 on tumor cells, activating the immune microenvironment within the tumor, increasing the number of effective CD8^+^ T cells, reducing the number of MSDCs and Treg cells, and effectively eliminating senescent tumor cells. Therefore, B68 can both induce tumor cell decay and inhibit tumor growth by regulating the immune system.

In the context of cancer treatment, the relationship between cellular senescence and tumor development is complex and multifaceted.^[^ ^38^, ^39^ ^]^ On the one hand, cellular senescence can be considered a mechanism for tumor suppression. For instance, the activation of oncogenes within cells may trigger a state known as oncogene‐induced senescence (OIS), in which cells enter an irreversible state of growth arrest, thus preventing the formation of tumors.^[^ ^40^, ^41^ ^]^ Consistent with our findings, B68 significantly upregulated the expression of p21 and p53 when acting on colorectal cancer cells, whereas B68 treatment significantly increased the percentage of β‐gal‐positive senescent cells in tumor tissue, causing cycle arrest. On the other hand, senescent cells influence the surrounding microenvironment by secreting the SASP, which contains proinflammatory cytokines, growth factors, and enzymes that may facilitate tumor invasion and metastasis, as well as promote tumor angiogenesis, supplying the necessary nutrients and oxygen for tumor growth.^[^ ^42^, ^43^ ^]^ Our research revealed that after B68 treatment, the levels of SASP‐related factors, such as IL6, GM‐CSF, CXCL3, and AREG, in colorectal cancer cells also significantly increased, indicating a certain degree of risk of immune suppression. Manuel Serrano's team has shown that chemotherapy can induce the senescence of cancer cells within the tumor, and PD‐L2 is highly upregulated in different types of cancer cells when they are induced to become senescent. Senescent cancer cells can protect themselves from the immune system by upregulating PD‐L2, while also recruiting immunosuppressive cells, creating an inhibitory environment that weakens the ability of lymphocytes to kill cancer cells. When senescent cells are cleared by immunotherapy, chemotherapy becomes more effective, providing a therapeutic strategy for the combination of chemotherapy and anti‐PD‐L2.^[^ ^44^ ^]^ Moreover, some studies have reported that PD‐L1 is heterogeneously expressed in senescent cells and that PD‐L1‐positive senescent cells accumulate in vivo with aging, which can further prevent clearance by the immune system.^[^ ^24^ ^]^ Previous studies have shown that berberine can suppress the deubiquitinating enzyme CSN5, reduce the expression of PD‐L1 on the surface of tumor cells, activate CD8^+^ T cells in the tumor microenvironment, and exert a tumor‐clearing effect.^[^ ^30^ ^]^ The berberine derivative B68 in this study, similar to berberine, can also reduce the expression of PD‐L1 on tumor cells and exert an antitumor immune effect. Therefore, our cancer treatment strategy involves inducing senescence in tumor cells and simultaneously reducing PD‐L1 expression on the surface of these senescent cells. This dual approach aims to activate the immune system to eliminate senescent cells without toxic side effects. Compared with the combination of chemotherapy and PD‐L2 antibodies, our strategy offers significant advantages, such as avoiding the harm caused by secondary medications and the potential side effects associated with chemotherapy and PD‐L2 antibody treatments.

BMI1, known as B‐cell specific Moloney murine leukemia virus integration site 1, is part of Polycomb repressive complex 1 (PRC1) and plays a central role in regulating gene silencing and chromatin structure, which is crucial for the self‐renewal of stem cells.^[^ ^45^, ^46^ ^]^ In this study, we predicted and verified BMI1 as a potential target of B68 via DARTS/MS, pull‐down, CETSA, and MST methods. During cellular senescence, the expression of BMI1 is often reduced, affecting cell cycle arrest and the production of the SASP.^[^ ^47^ ^]^ Next, we knocked down BMI1 in the colorectal cancer cell lines RKO and HCT116, and upon knockdown, the cells exhibited features of senescence, including upregulated expression of p53 and p21, indicating that downregulation of BMI1 significantly promoted cellular senescence. Additionally, B68 treatment did not induce further senescence in RKO cells with BMI1 gene knockout, indicating that B68 induces senescence through a BMI1‐dependent pathway.

Transcriptome sequencing of B68‐induced senescent cells revealed significant upregulation of antigen presentation mechanisms related to MHC‐I molecules, including the main transcriptional regulatory factor Nlrc5, in senescent cells. This finding suggested that B68‐induced senescence may increase the immunogenicity of tumor cells. It has been reported that the use of senescent cancer cells as vaccines can stimulate antitumor protective effects.^[^ ^48^, ^49^ ^]^ We demonstrated the ability of B68‐induced senescent MC38 cells to activate protection against subsequent rechallenge with untreated MC38 cells. As expected, B68‐induced senescent cells activate immune cells, including antigen‐presenting cells and CD8^+^ T cells, to provide specific immune protection against tumors. The advantage of this vaccine lies in the enhanced immunogenicity of senescent cells, which can more effectively activate the adaptive immune response, thereby resulting in a significant inhibitory effect on tumor growth in the MC38 animal model.

In summary, our research demonstrated that B68 possesses a dual mechanism of action in combating tumorigenesis. It facilitates tumor cell senescence, thereby inhibiting tumor cell proliferation, and simultaneously triggers an immune response by modulating the expression of PD‐L1 on senescent cells through the CSN5 pathway. This dual action not only targets the direct elimination of senescent tumor cells but also harnesses their potential as a therapeutic vaccine. Our findings offer novel insights and therapeutic strategies that could revolutionize the treatment of colorectal cancer by integrating senescence induction with immune modulation.

## 4 Experimental Section

### Cell Lines and Culture

Colorectal cancer cells (RKO, HCT116, CT26, LOVO, and SW620) were purchased from the Cell Bank of Shanghai Institute of Cell Biology, Chinese Academy of Sciences. MC38 cells were purchased from American Type Culture Collection (ATCC). The colon normal immortalized epithelial cell line NCM460 was obtained from InCell (San Antonio, TX) and cultured according to the manufacturer's instructions. Jurkat cells were donated by Kongming Wu's research group, Department of Oncology, Tongji Medical College, Huazhong University of Science and Technology (Wuhan, China). RKO cells were cultured in MEM; MC38 and HCT116 cells were cultured in DMEM and McCoy's 5A medium; LOVO cells were cultured in F‐12K medium; CT26 and NCM460 cells were cultured in RPMI‐1640; and SW620 cells were cultured in L15 medium. All media were purchased from Meilun (Dalian, China). Then, 100 mg mL^−1^ streptomycin (Meilunbio, China), 100 U mL^−1^ penicillin (Meilunbio, China) and 10% fetal bovine serum (Biological Industries, Cromwell, CT, USA) were added to the media, and the cells (excluding SW620 cells cultured in an air incubator) were placed in an incubator at 37 °C with 5% CO_2_.

### Mice

Female or male C57BL/6J mice and BALB/c nude mice, 6–8 weeks old, weighing 18–20 g, were purchased from Shanghai Jihui Laboratory Animal Breeding Company Limited (Shanghai, China) and housed at the Shanghai University of Traditional Chinese Medicine (SUTCM) Animal Center for animal experiments. The animals were housed in an SPF environment with 45% humidity, constant temperature (23 ± 2°C), and 12 h of light per day, and their food and drinking water were routinely sterilized by autoclaving.

### Antibody

All antibodies used are listed in Table S1 (Supporting Information).

### Cell Viability Assay

The cells were inoculated in 96‐well plates (Corning, USA) at a density of 8 × 10^3^ per well, and after the cells had adhered, the drug concentration was adjusted to the concentration required for the experiment. The drug‐containing medium was discarded, and 100 µL of CCK‐8 solution (Meilunbio, China) was added after 24 h. The cells were incubated at 37 °C for 1–4 h in the dark. The absorbance value (OD value) was measured at 450 nm by using an enzyme marker (BioTek, USA) to calculate the cell viability. Afterward, the absorbance value (OD value) was measured at 450 nm via an enzyme labeling instrument (BioTek, USA), and the cell viability was calculated.

### Apoptosis Assay

The cells in the logarithmic growth phase were inoculated in 6‐well plates at a density of 5 × 10^5^ per well, and after the cells had completely adhered, the drug was added for 24 h at the corresponding concentration. The cells were subsequently digested with trypsin, collected in centrifuge tubes, washed twice with PBS (Meilunbio, China), and stained with an Annexin V‐FITC/PI apoptosis double staining kit (BD Biosciences, USA). Then, 1× binding buffer was used to dilute Annexin V‐FITC and PI to 100 µL well^−1^, which were mixed thoroughly and incubated for 15 min at room temperature in the dark. Three hundred microliters of 1 × binding buffer was added before detection, and the mixture was filtered through a cell filtration sieve to prevent cell clumps from clogging the instrument. The results were detected via flow cytometry (Beckman Coulter Cytoflex, USA), and the data were processed and analyzed via FlowJo software.

### Cell Cycle Analysis

The logarithmic growth phase cells were digested into a cell suspension and inoculated in 6‐well plates at a density of 5 × 10^5^ per well. After the cells had adhered, the appropriate concentration of drugs was added, and the cells were treated for 24 h. All the cells were collected in centrifuge tubes and centrifuged at 300 × g for 5 min. The supernatant was discarded, and the cells were washed once with PBS buffer, mixed and fixed by adding 70% ice‐cold ethanol and left at 4 °C overnight. For detection, the ethanol was discarded, the samples were washed once with PBS, prepared pyridinium iodide staining solution (Beyotime, China) was added, and the samples were incubated for 30 min at 37 °C in the dark. The results were subsequently detected via flow cytometry (Beckman Coulter Cytoflex, USA) with a maximum excitation wavelength of 488 nm. The data were analyzed via ModFit LT 5.0.

### EdU Cell Proliferation Experiment

Cells in the logarithmic growth phase were inoculated into 96‐well plates at 8 × 10^3^ cells well^−1^. After the cells had adhered, drugs at the corresponding concentrations were added. The experiment was carried out according to the instructions of the EdU test kit (Beyotime, China). Finally, Operetta CLS High Content Cell Imaging Analysis (PerkinElmer, USA) was used to photograph and record the statistical data (the maximum excitation wavelength of Azide 488 was 495 nm, and the maximum emission wavelength was 519 nm).

### Cell Colony Formation Experiment

After digestion of the cells in the logarithmic growth phase, the cells were resuspended, 400 cells per well were inoculated in a 12‐well plate, and the culture was continued for 14 days or until the number of cells in most single clones was greater than 50. The medium was changed every 3 days during the process, and the cell status was observed. After cloning, the cells were washed with PBS once, 0.5 mL of 4% paraformaldehyde was added to each well for 30 min, and the cells were washed with PBS 3 times. Each well was stained with 0.5 mL of crystal violet solution for 15 min, then washed with PBS buffer and dried. Colonies of more than 10 cells were counted under a microscope to calculate the colony formation rate.

### Cell Activity Assay

The logarithmic growth phase cells were inoculated in a 96‐well plate at 8 × 10^3^ cells per well, and after the cells had adhered, the corresponding concentration of drugs was added. The culture medium was aspirated, and the cells were washed once with PBS. Then, 100 µl of Calcein AM assay working solution (Beyotime, China) was added to each well, the plates were incubated for 30 min at 37 °C in the dark, and photos were taken for recording via an Operetta CLS high‐content cell imaging system.

### Cell Scratch Experiment

A total of 6 × 10^5^ cells were inoculated in a 6‐well plate, and the cell confluence reached 100% after overnight culture. On the second day, a vertical scratch was made with a 20 µL tip. After the scratch was completed, the cells were washed 3 times with sterile PBS to remove the nonadherent cells, and the medium was then replaced with fresh serum‐free medium. The cells were placed in a 5% CO_2_ incubator at 37 °C. After 16 h, the cells were removed, the width of the scratches was observed and measured with a microscope, and photos were taken. The distance between the cells was statistically analyzed with ImageJ software.

### SA‐β‐Gal Activity Detection

The cells in good condition were inoculated in 12‐well plates. After the cells had adhered, they were incubated with different concentrations of drugs for 2 days, and the drug‐containing medium was removed and replaced with complete medium for 5 days. After 5 days, the culture plate was removed, the plate was washed with PBS, 1 mL of SA‐β‐gal staining fixing solution was added, and the plate was fixed at room temperature for 15 min. The fixing solution was removed, and the plate was washed with PBS. SA‐β‐gal dye solution (Beyotime, China) was prepared according to the instructions, and 1 mL was added to each well. The 12‐well plates were sealed with a parafilm membrane (PARAFILM, USA) to prevent evaporation and incubated at 37 °C overnight.

### Western Blot and Immunoprecipitation

The cells were inoculated into a 6‐well plate at a density of 5 × 10^5^ per well. After the cells had adhered, they were treated with different drug concentrations for 2 days, after which the cells were collected. The collected cells were centrifuged for 3 min at 400× g. After the media were discarded, the cells were washed twice with PBS and lysed on ice for 15 min. The supernatant was collected, and the protein concentration was determined via the BCA (Beyotime, China) method. Then, 5 × sample buffer was added, mixed, placed in a metal bath, and heated at 95 °C for 10 min. The corresponding loading amount was calculated, and SDS‐PAGE was performed. After electrophoresis, the proteins were transferred to a PVDF membrane (Millipore, USA). After membrane transfer, the membrane was incubated with 5% skim milk powder at room temperature for 1 h. Primary antibody was added, and the membrane was incubated overnight at 4 °C. The membrane was then incubated with secondary antibody at room temperature for 1 h, after which images were taken with an imaging system (Bio‐Rad, USA).

For the inhibitor experiments, the cells were treated with B68 in the absence or presence of the proteasome inhibitor MG132 (133407‐82‐6, MedChemExpress, USA) or the lysosome inhibitors bafilomycin (Baf, 88899‐55‐2, MedChemExpress, USA), chloroquine (CQ, 54‐05‐7, MedChemExpress, USA), and 3‐methyladenine (3‐MA, 5142‐23‐4, MedChemExpress, USA). For cycloheximide (CHX, 66‐81‐9, MedChemExpress, USA) chase studies, cells were treated with B68 for 16 h, and 25 µg ml^−1^ CHX was added at the indicated time points. The subsequent processing was consistent with the above procedure.

Protein ubiquitination was analyzed by immunoprecipitation. After transfection for 48 h, the cells were collected, an appropriate amount of precooled cell lysis buffer (including protease inhibitor) was added (MedChemExpress, China), and the cells were lysed on ice for 30 min. The cells were then centrifuged at 13 500 × g at 4 °C for 30 min to obtain the supernatant of the lysate. IgG or PD‐L1 antibody was added to the supernatant of the lysate, which was subsequently incubated with rotation at 4 °C overnight. Then, 30 µL of protein A/G beads (Santa Cruz Biotechnology, USA) were added and incubated at 4 °C for 3 h. Then, 500 µL of IP lysate was added to rinse the magnetic beads, which were then washed 5 times. Then, an equal volume of 2× loading buffer was added to the magnetic beads, the mixture was heated at 95 °C for 10 min in a metal bath, and the samples were stored in a ‐20 °C refrigerator.

### RNA Extraction and Real‐Time PCR Analysis

Total RNA was isolated from the collected cells via RNAiso Plus (Takara, Shiga, Japan), followed by reverse transcription of total mRNA (2 µg) into cDNA via a Prime Script RT reagent kit (Yeasen; Shanghai, China). Quantitative PCR was performed via SYBR Green (Roche, Mannheim, Germany) and a LightCycler96 qPCR system (Roche, Mannheim, Germany). The relative expression of target genes was calculated via the ^△△^CT method. The sequences of the primers used are shown in Table S2 (Supporting Information).

### Detection of Membrane Proteins by Flow Cytometry

After the cells were treated with different concentrations of drugs, they were collected, washed twice with PBS, and incubated with an anti‐PD‐L1 antibody at 4 °C for 30 min in the dark. After incubation, the contaminated solution was centrifuged and discarded. The cells were washed and suspended in PBS. The expression of PD‐L1 was detected via flow cytometry (Beckman Coulter Cytoflex, USA).

### Immunofluorescence Staining Assay

After 24 h of treatment, the media in the 12‐well plates were discarded, the cells were washed twice with PBS buffer, and then the cells were fixed for 15 min with 4% paraformaldehyde. Then, the cells were washed 3 times with PBS and incubated with 5% BSA at room temperature for 1 h. Then, the primary antibody was added, and the cells were incubated overnight on a shaking bed at 4 °C. Then, the fluorescently labeled secondary antibody was added, and the samples were incubated at room temperature for 1 h in the dark and washed with 5% BSA (Beyotime, China) 3 times. An anti‐fluorescence quencher containing DAPI (Beyotime, China) was added before loading, and the expression level of PD‐L1 on the surface of the cell membrane was detected with a Cytation 5 instrument (BioTek, USA).

### T Cell‐Mediated Tumor Cell Killing Assay

RKO and HCT116 cells were inoculated in 12‐well plates at a density of 3 × 10^5^ per well, and after the cells had adhered, different concentrations of drugs were added and incubated for 24 h. Then, activated T cells (Jurkat cells stably transfected with human PD‐1) were added for coculture with the tumor cells. T cells were cocultured for 24 h at a ratio of tumor cells:T cells = 1:8. After 24 h, the cells were washed twice with PBS buffer, fixed with 4% paraformaldehyde (Meilun Biotechnology, China), and discarded. After 24 h, the T cells were washed twice with PBS buffer, fixed with 4% paraformaldehyde (Meilun Biotechnology, China), discarded, washed three times with PBS buffer, stained with 0.5 mL of crystal violet staining solution for 15 min, washed with PBS buffer, and then imaged with Cytation 5. The area of the remaining cells was calculated via ImageJ. The cells were then stained with 0.5 mL of crystal violet staining solution for 15 min.

### Proteomic Analysis

After drug treatment, RKO cells were subjected to the SDT lysis method, the supernatant was removed, and the mixture was subjected to enzyme digestion with trypsin (Promega Corporation, USA) at 37 °C for 16–18 h. The collection tube was replaced with a new tube at the end of enzyme digestion, 50 µL of TEAB (100 mm) (Thermo Fisher Scientific, USA) was added, and the mixture was centrifuged to collect the filtrate. The filtrate was collected by centrifugation. The peptides were desalted via a C18 cartridge (Shimadzu GL, Japan), and the peptides were lyophilized and redissolved via the addition of 0.1% formic acid (Thermo Fisher, USA) for BCA (Beyotime, China) quantification. The TMTpro 16plex (Thermo Fisher Scientific, USA) label was reconstituted with acetonitrile (Thermo Fisher Scientific, USA), added to the samples, and incubated for 1 h in the dark, followed by the addition of 5% hydroxylamine (Thermo Fisher Scientific, USA) to terminate the reaction. The samples were combined and dried under vacuum using a centrifugal concentrator. The samples were then desalted via a C18 cartridge; the samples were then graded and analyzed by LC‐MS/MS.

### Drug Affinity Target Stabilization Tests (DARTS)

The basic principle of DARTS is that the binding of small molecule ligands to target proteins can stabilize the target proteins and increase their resistance to hydrolysis by protein hydrolases. After the proteins bind to the small molecule drug, the mixed protein after the affinity reaction was enzymatically hydrolyzed, and the enzyme hydrolysis product was separated by gel electrophoresis. Compared with the blank group, there was an increase in the protein fragments bound to the drug. The prepared protein samples were divided equally. Different concentrations of B68 were added, and an equal volume of DMSO was added to the control group. Protease hydrolysis was subsequently carried out to detect differences between the control group and the treatment group proteins by immunoblotting and Coomassie brilliant blue staining; the samples were prepared by the collection of the gel bands of the target proteins, which were analyzed by LC‐MS/MS.

### Pull Down the Experiment

The prepared cell lysate was divided evenly and incubated on a rotator at 4 °C for 4 h with Bio, Bio‐B68, 10 × B68 or 20 × B68. Then, magnetic beads (Sigma, USA) were added and incubated at 4 °C for 4 h, centrifuged at 1000× g at 4 °C for 3 min, and washed with IP lysis buffer (Beyotime, China) 5 times to prepare protein samples. Electrophoresis was carried out, and the obtained gel was silver stained, cut and decolorized. The samples were processed according to the proteomics sample preparation method and analyzed by LC‐MS/MS.

### Molecular Modeling

The amino acid sequence of BMI1 was sourced from UniProt. The 3D structure of BMI1 was predicted via AlphaFold and Molecular Operating Environment (MOE), and a molecular docking model of B68 with the 3D structure of BMI1 was constructed in the MOE. Regularized proteins were used to identify important amino acids in the predicted binding pockets. After energy minimization via the prepared ligand protocol, all conformations of B68 were induced to fit into the selected active sites via the MOE DOCK module. The docking compounds were scored on the basis of their binding patterns at the binding sites.

### MST Experiment

A plasmid carrying the GFP target protein was purchased from Guannan Biology (Hangzhou, China), and sequence information for the target protein was obtained from NCBI. The BMI1‐GFP and CSN5‐GFP plasmids were cis‐transfected into 293T cells for expression, and the cells were collected after 48 h. The supernatant was collected after lysis. The compounds were mixed with the supernatant separately to prepare sixteen gradient concentrations to be tested, and the mixed samples were aspirated using a capillary tube and then detected with a MonolithTM NT.115 MST (NanoTemper, Germany). The mass action equation in NanoTemper software was applied to calculate the Kd value.

### Cell Transfection Experiments

siRNAs targeting BMI1, UBAP2L, YBOX1, and S100A9 were purchased from GenePharma (Shanghai, China), and the sequences used are listed in Table S3 (Supporting Information). A negative control (NC) was used as a control. After the RKO cells had adhered, the double‐stranded siRNAs were transfected with Liposome 2000 (Invitrogen, Carlsbad, CA), and the medium was replaced with fresh medium after 6–8 h of transfection. After 48 h, the cells were subjected to secondary transfection, and after incubation, β‐galactosidase staining was performed. The plasmids used for transfection were pcDNA3.1‐Ub and VP048 pCMV‐MCS‐ 3*Flag‐SYVN1, which were purchased from GenePharma (Shanghai, China). The other steps and reagents used were similar to those used for siRNA transfection. BMI1‐deficient RKO and HCT116 cells were generated by targeting the sgRNA sequence of BMI1 via the lentivirus‐Cas9 system. The sgRNA sequences used are shown in Table S3 (Supporting Information).

### Cell Thermal Shift Assay

RKO cells were collected, lysed with IP lysis buffer (Beyotime, China) for 30 min at 13 500× g, and centrifuged for 15 min. The supernatant was collected, divided equally into two portions, and then treated with 200 µm B68 and an equal volume of DMSO. The plates were rotated and incubated for 1 min at room temperature. Each sample was divided into 100 µL, heated at different temperatures (37 °C, 62 °C, 65 °C, 68 °C, 71 °C, 74 °C, 77 °C, 80 °C, and 83 °C) for 3 min, and then cooled to room temperature. After centrifugation at 4 °C and 13 500 × g for 15 min, the supernatant was collected and prepared for SDS‐PAGE analysis.

### Assessment of BMDC Antigen Capture

The femurs and tibias of C57BL/6J mice were harvested, and the bone marrow was isolated, rinsed, and centrifuged at 400× g for 5 min. The bone marrow was incubated with red blood cell lysis buffer for 5 min, after which the cells were sequentially filtered through 100 and 70 µm meshes to remove clumps. A total of 5 × 10^4^ cells per well were seeded in a 6‐well plate and cultured in RPMI 1640 medium supplemented with mouse‐derived GM‐CSF (25 ng ml^−1^, Peprotech 315‐03) and IL‐4 (10 ng ml^−1^, Peprotech 214‐14). On days 2 and 4, 3/4 of the medium was replaced, and the medium was replenished with GM‐CSF and IL‐4. On day 6, the dendritic cells (DCs) were harvested and replated to promote further maturation. On day 10, the dendritic cells were collected and cocultured with senescent cancer cells at a 1:1 ratio for 24 h. The expression of DC activation and maturation markers was subsequently analyzed via flow cytometry.

### Animal Experiment

All 6–8 weeks old C57BL/6J mice and BALB/c nude mice weighing 18–20 g were purchased from Shanghai Jihui Laboratory Animal Breeding Company Limited (Shanghai, China) and were generated following the ethical obligations of the Department of Laboratory Animal Science, Shanghai University of Traditional Chinese Medicine. MC38 cells in the logarithmic growth phase were counted by resuspending the cells in saline. A total of 8 × 10^5^ cells were inoculated subcutaneously into C57BL/6J mice. When the average tumor volume reached 50 mm^3^, the mice were randomly divided into different groups: blank group, control group, and B68 and B1 treatment groups. The treatment group was injected with 8 mg kg^−1^ B68 per day, and the control group was injected with the same volume of saline. Mouse body weight and tumor size were recorded every two days.

The axilla of the BALB/c nude mice were inoculated with RKO and HCT116 cells (10 × 10^5^ cells), as described above, and the mice were randomly divided into three groups: control group, B68 treatment group, and B1 treatment group. Mice in the treatment group were injected with 8 mg kg^−1^ B68 or 8 mg kg^−1^ B1 daily. Mice in the control group were injected with the same volume of saline. Mouse body weight and tumor size were recorded every two days.

For the combined treatment of B68 and CTLA4 antibodies, the mice were randomly divided into five groups as described above: control group (saline), PD‐1 antibody‐treated group, CTLA4 antibody‐treated group, B68‐treated group, B68 and CTLA4 antibody combination group, administration concentration PD‐1 antibody (100 µg/100 µL), CTLA4 antibody (100 µg/100 µL), B68‐treated group (8 mg kg^−1^). B68 was injected every day until the average tumor volume reached ≈50 mm3, and PD‐1 and CTLA4 antibodies were administered every five days. In addition, mouse body weight and tumor size were recorded every two days.

At the end of the experimental period, the tumor tissues of the mice were removed, photographed, recorded, and weighed. Tumor tissues from each group were analyzed by flow cytometry, immunohistochemistry, WB strips and β‐galactosidase staining, and the viscera were stained with H&E.

Two drugs, azoxymethane (AOM) and dextran sulfate sodium salt (DSS), were commonly used to establish classical mouse models of colitis carcinogenesis and can reflect the process of intestinal carcinogenesis more realistically. For the AOM/DSS animal model experiments, 6–8 weeks old C57BL/6J mice (male) were used. First, mice were induced via intraperitoneal injection of 12.5 mg kg^−1^ AOM (Sigma, St. Louis, MO). After one week of receiving normal drinking water, the mice were allowed to drink water containing 2.5% DSS (MP Biomedicals, USA) for one week to induce colitis. The water was then replaced with normal drinking water for two weeks. The above steps were repeated for three consecutive cycles (DSS drinking water‐normal drinking water) to mimic the cancerous process of human ulcerative colitis. During the third cycle, the concentration of DSS was adjusted from 2.5% to 2%. On day 65, the mice were sacrificed, and several indicators, including intestinal appearance, length, tumor incidence, and mouse body weight, were measured.

For the animal vaccine experiments, for prophylactic immunization against cancer cells, senescent cells were resuspended in PBS, and 1.5 × 10^5^ senescent cells were injected subcutaneously into the left side of 6–8 weeks old C57BL/6J mice. After seven days, 1.5 × 10^5^ cancer cells were injected subcutaneously into the animals, and mouse body weight and tumor size were recorded every two days (DELIXI ELECTRIC, China).

To analyze the adjuvant properties of the cancer cells, 6–8 weeks old C57BL/6J mice were subcutaneously injected with the carrier (PBS), and 1.5 × 10^5^ senescent cells were resuspended in 100 µL of PBS. After seven days, the subcutaneous inoculation sites were dissected, and histological images were analyzed.

### Clinical Tissue Samples

All patients were treated at Longhua Hospital, Shanghai University of Traditional Chinese Medicine (Shanghai, China). All samples were collected after written informed consent was obtained from patients and/or their parents. The study protocol was approved by the Ethics Committee of Longhua Hospital affiliated to Shanghai University of Traditional Chinese Medicine.

### Data Processing

The data were expressed as mean ± SD or mean ± SEM, with *P* < 0.05 considered to indicate statistical significance. The data were collated and analyzed using GraphPad Prism 8.0.1. Statistical analysis was performed using Student's *t* test for comparisons between two groups and one‐way ANOVA for comparisons among multiple groups. ImageJ was used for gray intensity quantification of protein bands and mean fluorescence intensity measurements, and FlowJo was used to analyze the flow correlation data. The data were analyzed with GraphPad Prism 8.0.1.

## Conflict of Interest

The authors declare no conflict of interest.

## Author Contributions

H.H., Q.W., D.Y., and X.T. contributed equally to this work. S.L., W.Z., B.L., and H.X. performed conceptualization, original draft, methodology, review and editing, funding acquisition and supervision. H.H., Q.W., D.Y., and X.T. analyzed the data, carried out the experiments, generated the figures and wrote the paper. M.G., H.X., S.T., Q.Z., M.X., X.G., H.Z., C.X., L.L., and S.X. participated in part of the experiments. K.C., W.Z., and X.‐W.L. performed conceptualization, methodology, and supervision.

## Supporting information



Supporting Information

## Data Availability

The data that support the findings of this study are available from the corresponding author upon reasonable request.
