# Supplementary material for: Berberine Derivative B68 Promotes Tumor Immune Clearance by Dual‐Targeting BMI1 for Senescence Induction and CSN5 for PD‐L1 Degradation
Source: Adv Sci (Weinh). 2024 Dec 25;12(7):2413122. doi: 10.1002/advs.202413122 (PMC11831439; doi:10.1002/advs.202413122)
Supplement: Supplementary file 1 — Supporting Information [file ADVS-12-2413122-s001.docx]

**Supplemental Information**


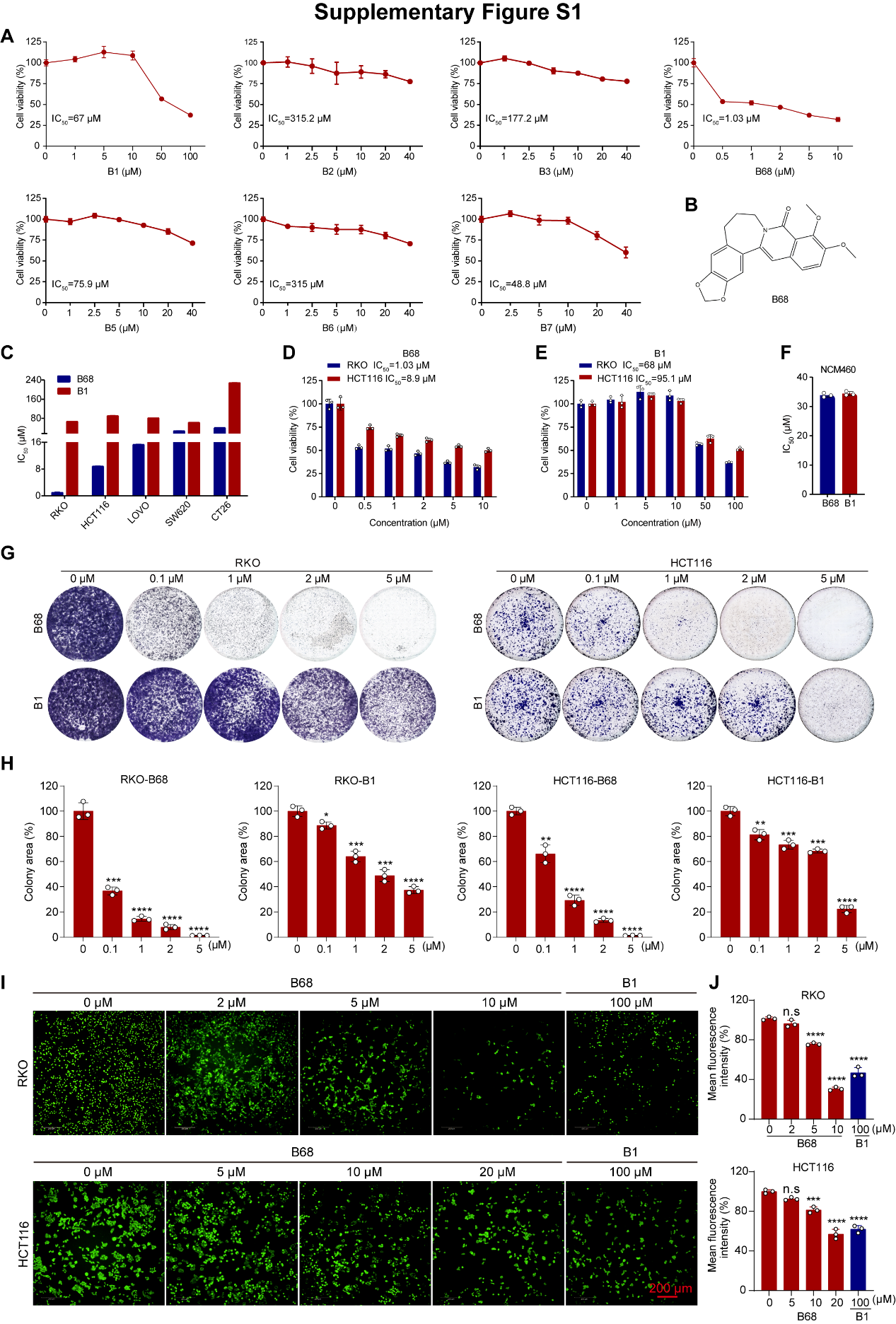


**Figure S1. Screening for anticolorectal cancer activity of the natural small molecule berberine derivative B68**

(A) B68 is the best berberine derivative for inhibiting RKO cells, as demonstrated by CCK-8 experiments (n = 3, error bars represent SEM, mean ± SEM). (B) Structural formula of B68. (C) The B68-sensitive cell lines selected for the CCK8 experiments were HCT116 and RKO cells. (D, E) The inhibition rates and semiinhibitory effects of B68 and berberine (B1) on RKO and HCT116 cells were detected via a CCK-8 assay (n = 3, error bars represent SEM, mean ± SEM). (F) Effects of the semiinhibitory concentrations of B1 and B68 on human normal intestinal epithelial cells (NCM460) (n = 3, error bars represent SEM, mean ± SEM). (G) The colony-forming ability of RKO and HCT116 cells treated with B68 was assessed via crystal violet staining. (H) Bar chart showing the number of groups in Figure S1G (n = 3, error bars represent SEM, mean ± SEM, Student’s t-test, compared with the control group). (I) Proliferation of RKO and HCT116 cells treated with B68 and berberine was detected via an EdU kit, as indicated by green fluorescence. Scale bars, 200 μm. (J) Quantitative results of Figure S1I as determined by EdU staining. (n=3, error bars represent SEM, mean ± SEM, one-way ANOVA, compared with the control group). n.s, not significant; *p < 0.05, **p < 0.01, ***p < 0.001, ****p < 0.0001.


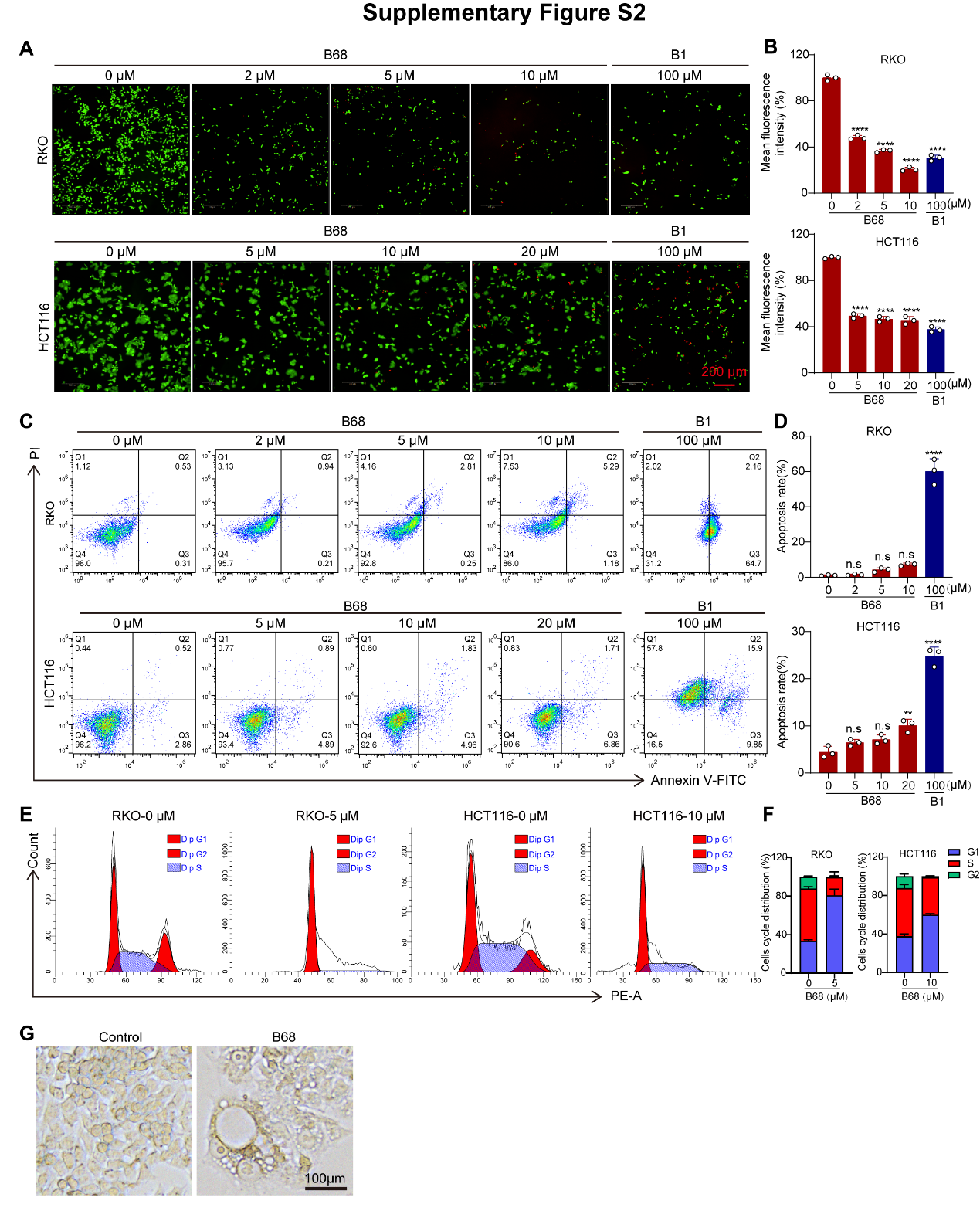


**Figure S2.** **Validation of the anticolorectal cancer activity of the natural small molecule B68**

(A) RKO and HCT116 cells were treated with B68 for 24 h, and cell viability was measured by calcineurin/PI staining (scale bar: 200 μm). (B) Results of the quantitative analysis of the data in Figure S2A. (n = 3, error bars represent SEM, mean ± SEM, one-way ANOVA, compared with the control group). (C, E) After RKO and HCT116 cells were treated with B68 for 24 hours, cell apoptosis and cell cycle progression were detected via flow cytometry. (D, F) Quantification of apoptosis and cell cycle data. (n = 3, error bars represent SEM, mean ± SEM, Student’s t-test). (G) Morphological changes in HCT116 cells after B68 treatment (scale bar: 100 μm). n.s, not significant; **p < 0.01, ***p < 0.001, ****p < 0.0001.


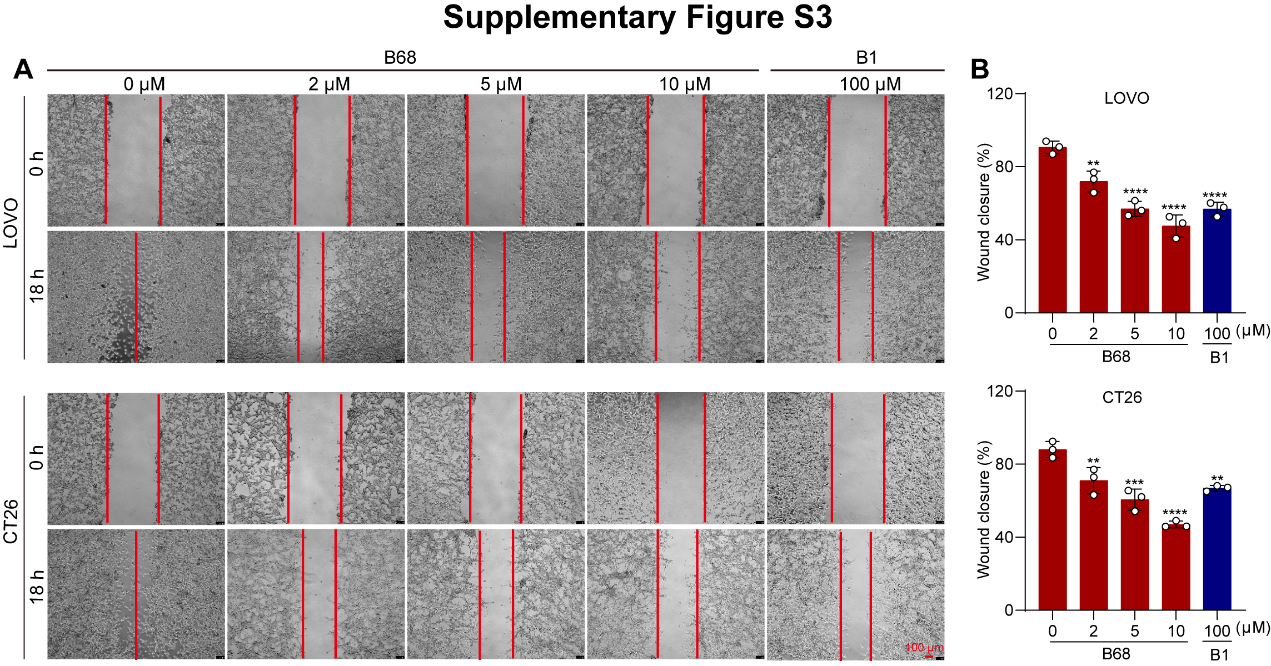


**Figure S3****. B68 can inhibit the migration of colorectal cancer cells**

(A) The migration ability of LOVO and CT26 cells after treatment with different concentrations of B68 was examined via the scratch method, and the results of the quantitative analysis are shown in (B). Scale bar: 100 μm (n = 3, error bars represent SEM, mean ± SEM, one-way ANOVA, compared with the control group). **p < 0.01, ***p < 0.001, ****p < 0.0001.


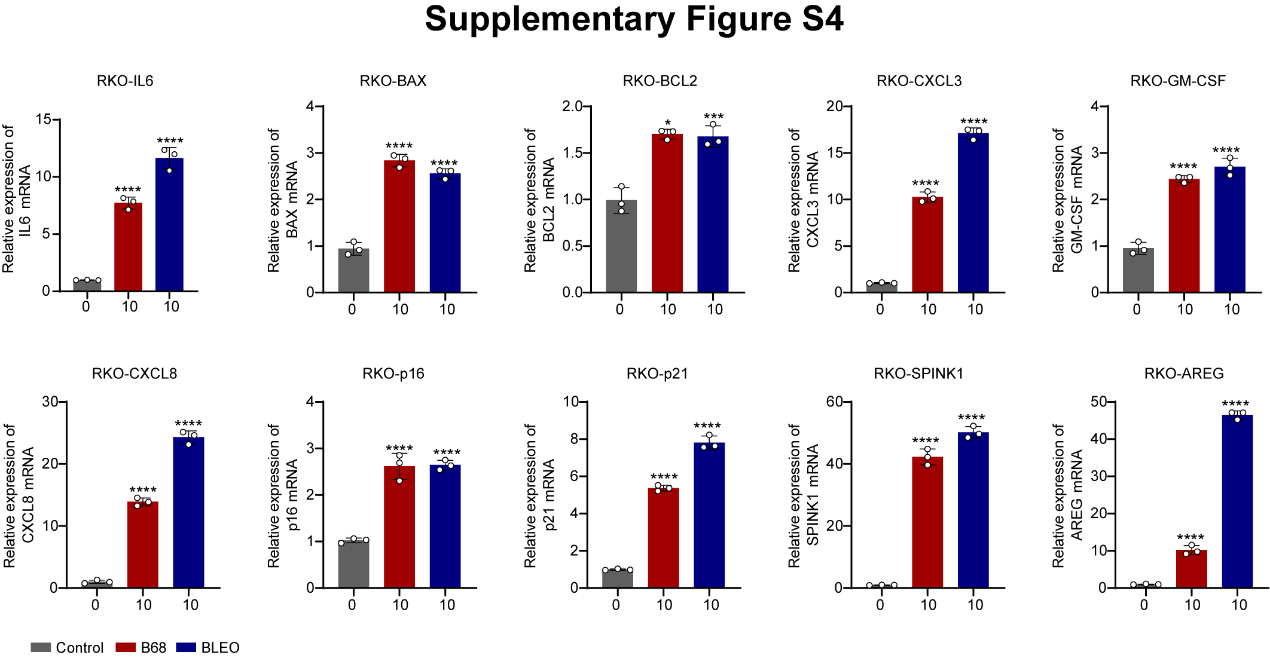


**Figure S4****. B68 upregulates SASP-related mRNA expression in RKO cells**

RKO cells were treated with B68 (10 μM) and bleomycin (10 mg/mL) for 7 days, and then, quantitative RT-PCR was conducted to determine the expression levels of senescence-associated secretory phenotype (SASP)-related mRNAs, such as IL6, BAX, BCL2, CXCL8, CXCL3, GM-CSF, p16, p21, SPINK1, and AREG (n = 3, error bars represent SEM, mean ± SEM, one-way ANOVA). *p < 0.05, ***p < 0.001, ****p < 0.0001.


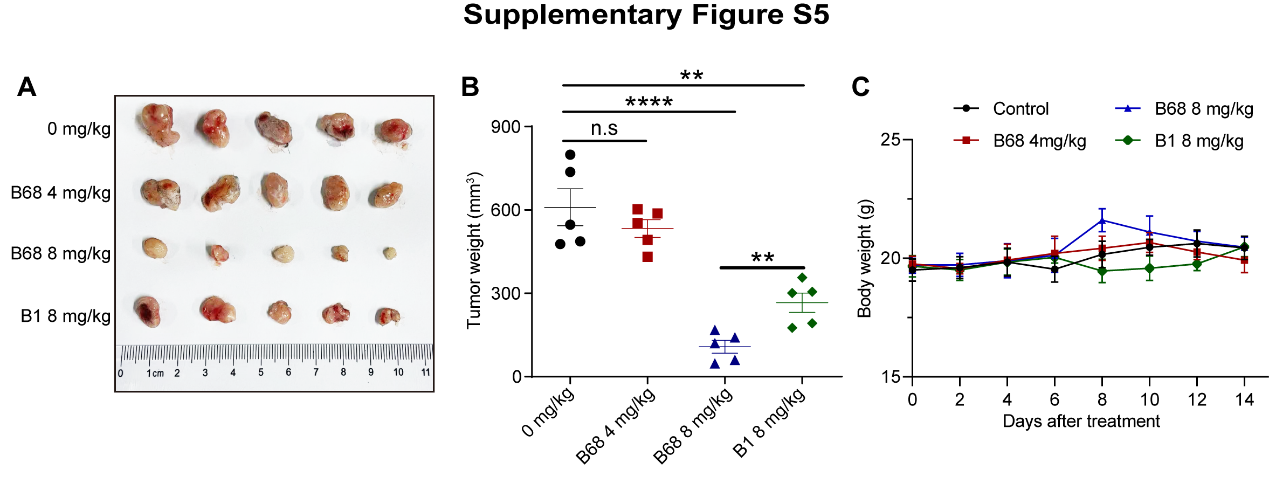


**Figure S5.** **B68 has** **significant antitumor effects on colorectal cancer**

(A) Representative images of MC38 tumor xenografts, (B) tumor weight, and (C) mouse body weight in C57BL/6J mice following a 14-day treatment with B68 at doses of 4 mg/kg and 8 mg/kg, as well as B1 at 8 mg/kg (n = 5, error bars represent SEM, mean ± SEM, Student’s t-test). n.s, not significant; **p < 0.01, ****p < 0.0001.


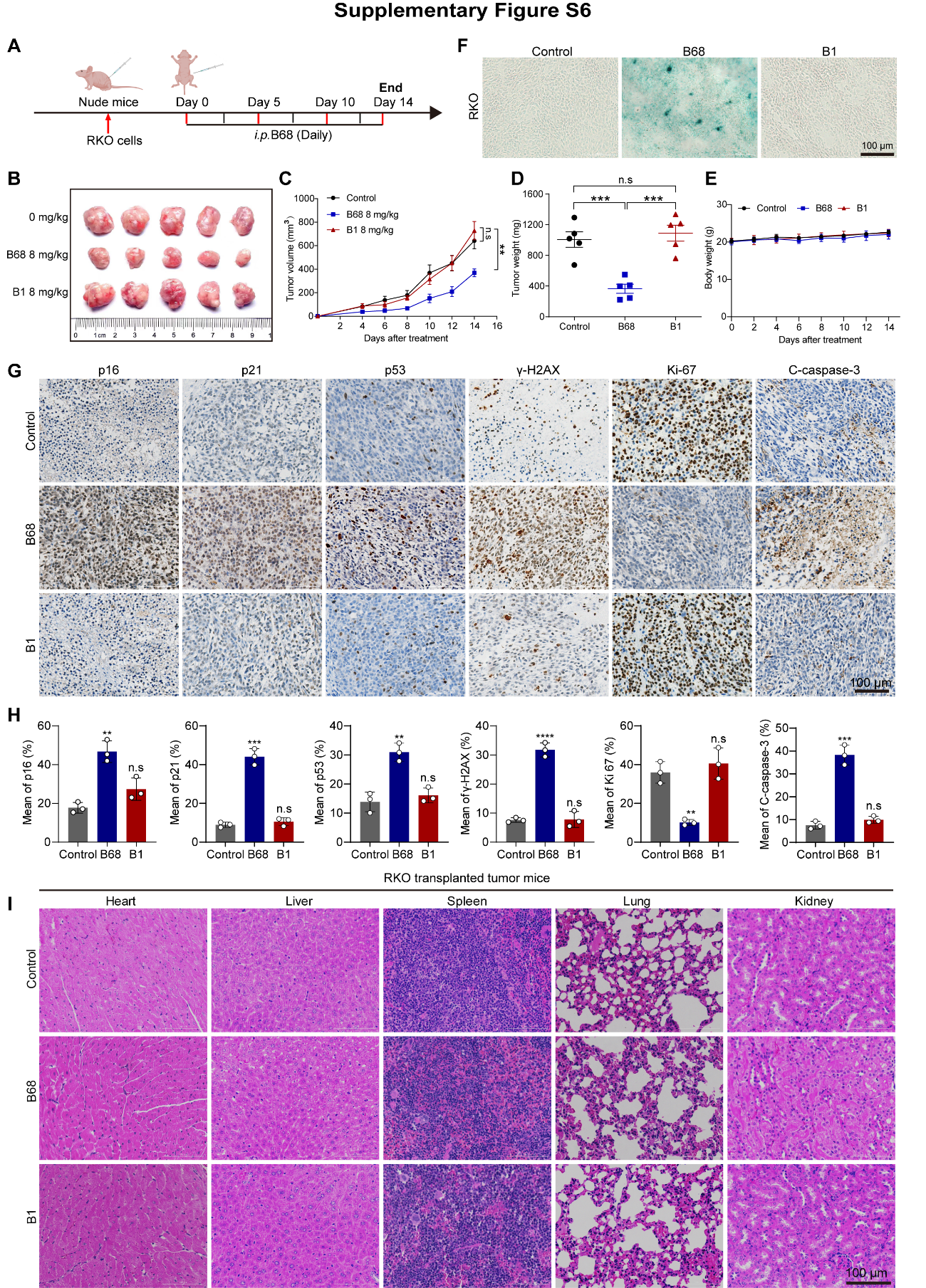


**Figure S6.** **B68 exerts antitumor effects by inducing senescence in colorectal cancer cells**

(A) Schematic diagram of the subcutaneous tumor model in BALB/c nude mice. Daily intraperitoneal injections of PBS, B68 (8 mg/kg), or B1 (8 mg/kg) were administered for a period of 2 weeks. (B) Representative images of RKO tumors, (C) tumor volume, (D) tumor weight, and (E) mouse body weight in BALB/c nude mice (n = 5, error bars represent SEM, mean ± SEM, Student’s t test). (F) At the end of drug administration, fresh tumors were removed, and frozen sections were prepared for the evaluation of *in vivo* senescence via SA-β-Gal staining; scale bar, 100 μm. (G) Representative immunohistochemical (IHC) staining results of cleaved caspase 3, Ki-67, p16, p21, p53 and γ-H2AX in BALB/c nude mice from different treatment groups (Scale bar, 100 μm). (H) Quantitative graph of the immunohistochemical staining in Figure S6G (error bars represent SEM, mean ± SEM, Student's t-test). (I) Hematoxylin-eosin staining results of major organs in BALB/c nude mice. n.s, not significant; **p < 0.01, ***p < 0.001, ****p < 0.0001.


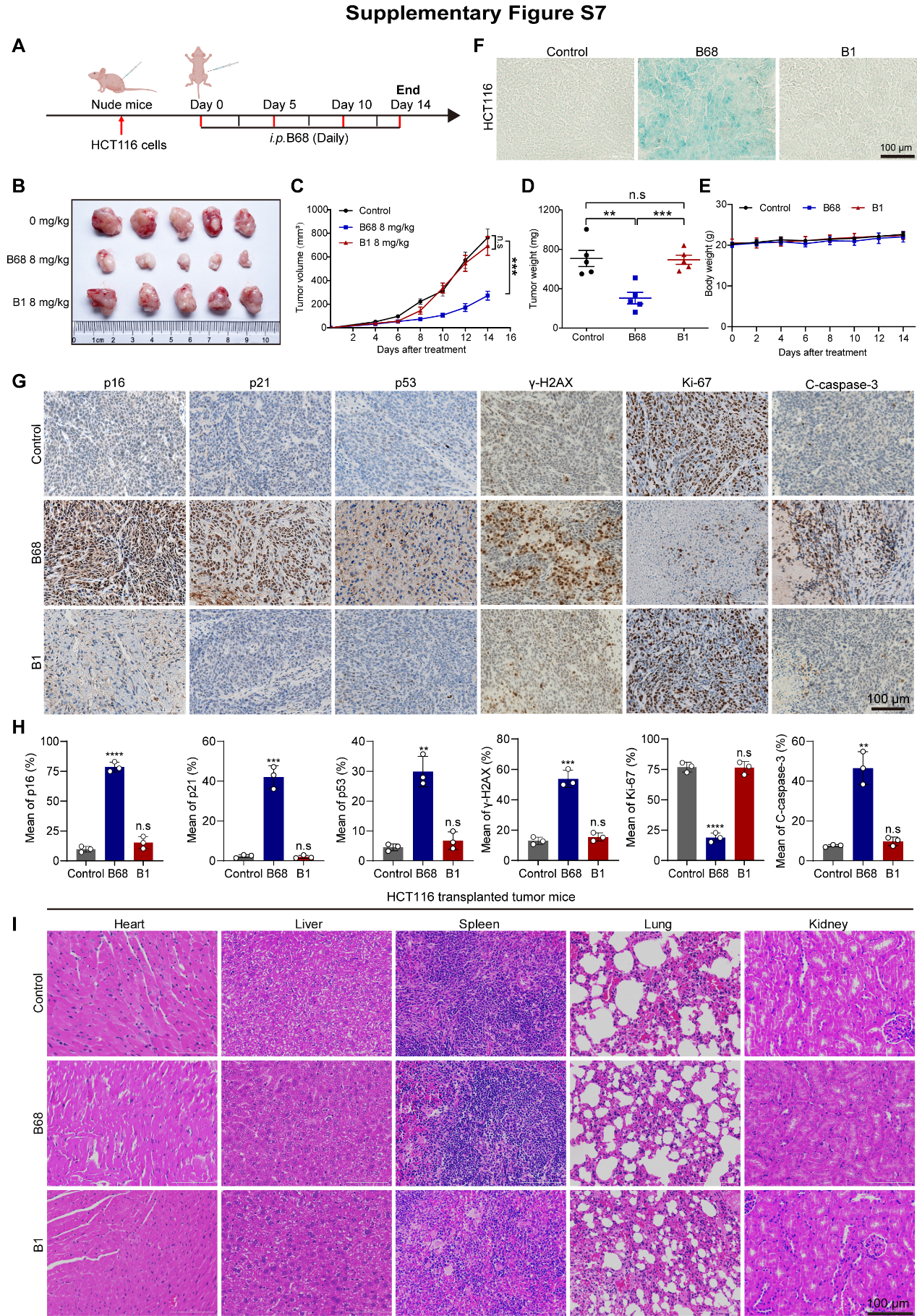


**Figure S7.** **B68 inhibits the growth of HCT116 xenograft tumors by inducing senescence**

(A) Schematic diagram of the subcutaneous tumor model in BALB/c nude mice. Daily intraperitoneal injections of PBS, B68 (8 mg/kg), or B1 (8 mg/kg) were administered for a period of 2 weeks. (B) Representative images of HCT116 tumors, (C) tumor volume, (D) tumor weight, and (E) mouse body weight in BALB/c nude mice. (n = 5, error bars represent SEM, mean ± SEM, Student’s t test). (F) SA-β-Gal staining of tumor tissues; scale bar, 100 μm. (G) Representative immunohistochemical (IHC) staining results of cleaved caspase 3, Ki-67, p16, p21, p53 and γ-H2AX in nude mouse tumor tissues. Scale bar, 100 μm. (H) Quantitative results of IHC staining of BALB/c nude mouse tumor tissues. (error bars represent SEM, mean ± SEM, Student’s t-test). (I) HE staining of tissue sections from different organs of each group of BALB/c nude mice. n.s, not significant; **p < 0.01, ***p < 0.001, ****p < 0.0001.


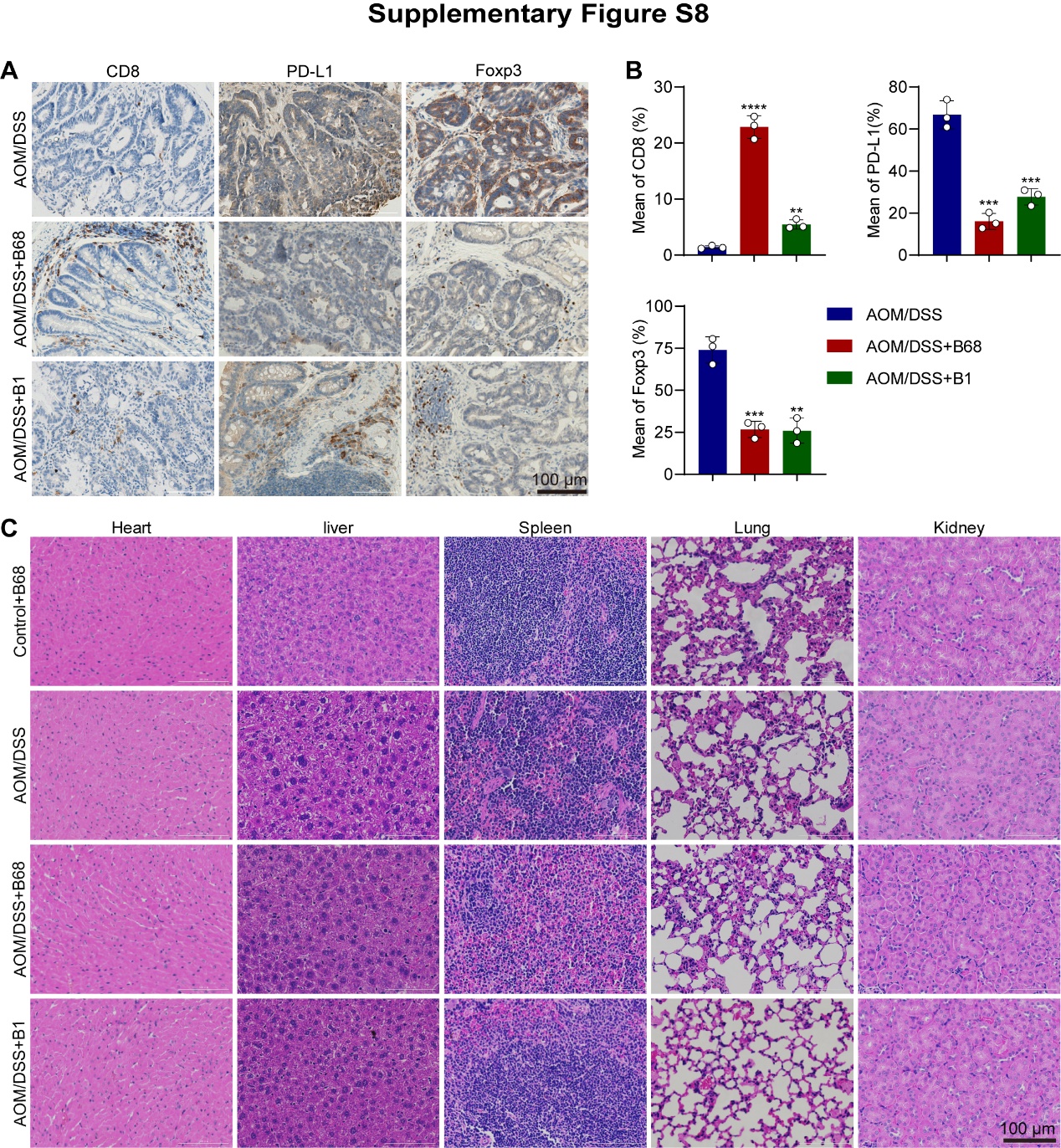


**Figure S8. B68 inhibited colorectal cancer induced by AOM/DSS**

(A) Representative immunohistochemical (IHC) staining results of CD8, PD-L1 and Foxp3 in mouse colon tumor tissues and quantitative analysis are shown on the right. (B) Scale bar, 100 μm (error bars represent SEM, mean ± SEM, Student's t-test). (C) H&E staining results of major organs in C57BL/6J mice. **p < 0.01, ***p < 0.001, ****p < 0.0001.


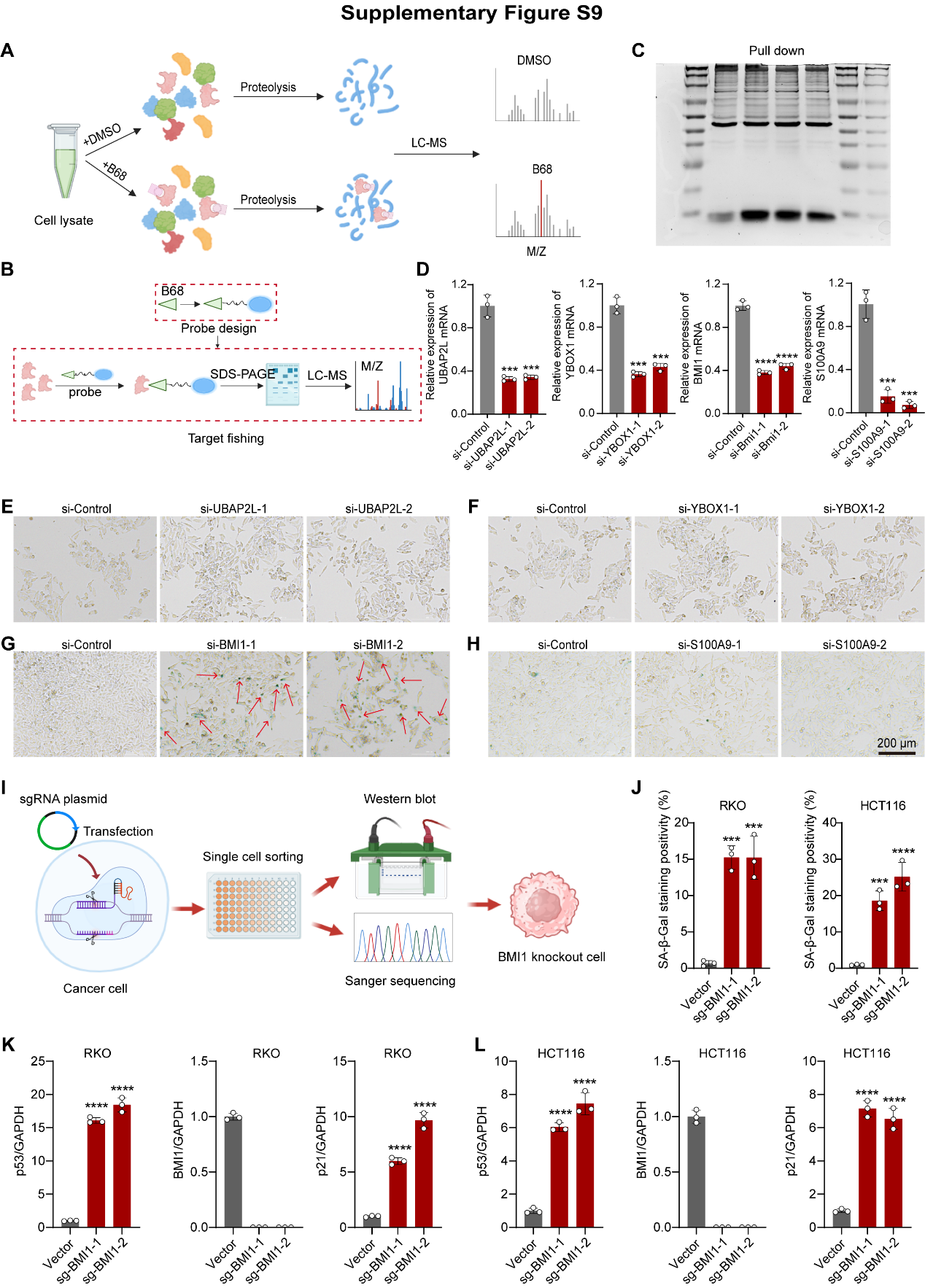


**Figure S9. DARTS****, pull-down predicts the potential target genes of B68-induced colorectal cancer cells**

(A) Flowchart of the DARTS experiment. (B) Flowchart of the pull-down experiment. (C) Results of SDS-PAGE analysis of the pulldown products. (D-H) After the potential target genes of B68, such as BMI1, S100A9, YBOX1, and UBAP2L, were silenced for one week, the knockdown efficiency of the siRNAs was assessed, and the results of SA-β-gal staining were examined. Scale bars, 200 μm (n = 3, error bars represent SEM, mean ± SEM, Student's t test). (I) Flowchart of the CRISPR/Cas9 gene knockout procedure. (J) Statistical analysis of the percentage of SA-β-gal-positive RKO and HCT116 cells after BMI1 gene knockout. Scale bars, 100 μm (n = 3, error bars represent SEM, mean ± SEM, one-way ANOVA). (K, L) Quantification of BMI1, p21 and p53 protein expression in RKO and HCT116 cells (n = 3, error bars represent SEM, mean ± SEM, one-way ANOVA). ***p < 0.001, ****p < 0.0001.


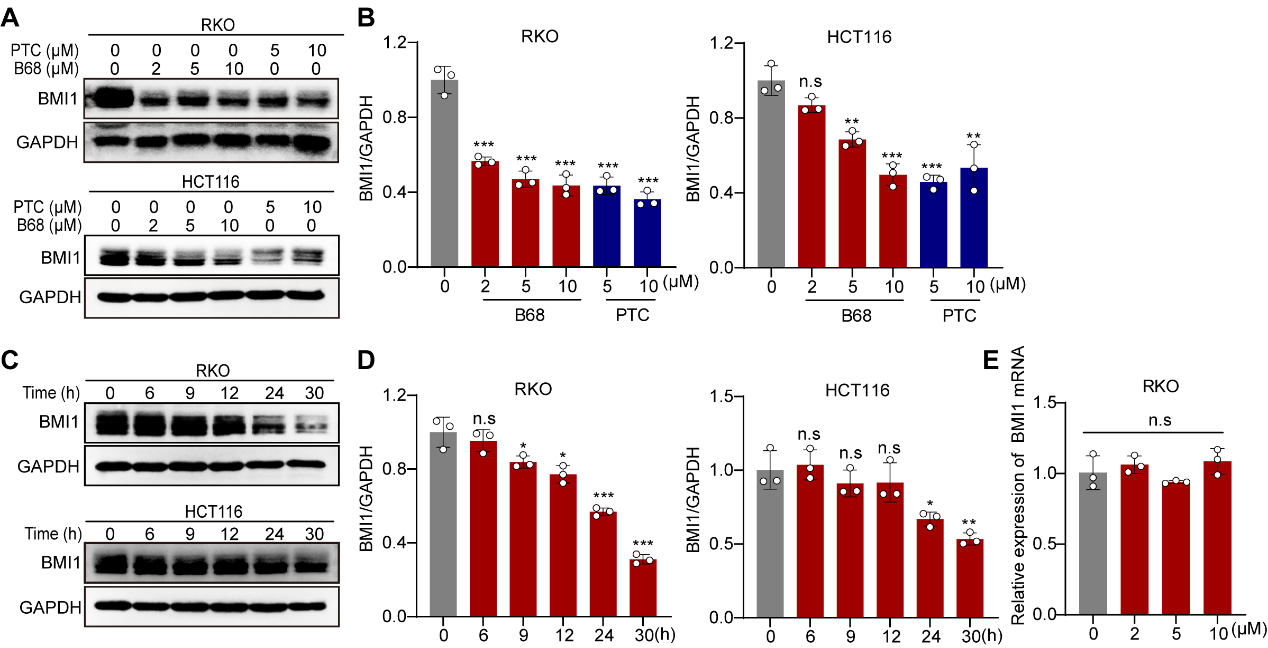


**Figure S10. B68 decreased BMI1 protein expression but had no effect on BMI1 mRNA levels**

(A) RKO and HCT116 cells were treated with different concentrations of B68 and the BMI1 inhibitor PTC209 for 30 h. Total BMI1 protein expression was detected by immunoblotting (IB). (B) Quantification of BMI1 protein levels is shown on the right (n = 3, error bars represent SEM, mean ± SEM, Student's t-test). (C) RKO and HCT116 cells were treated with B68 (10 µM or 20 µM) for the indicated times, and assays were performed to detect the total BMI1 protein level. (D) Quantification of BMI1 protein expression is shown on the right (n = 3, error bars represent SEM, mean ± SEM, Student's t-test). (E) Effects of different concentrations of B68 on BMI1 mRNA levels in RKO cells (n = 3, error bars represent SEM, mean ± SEM, Student’s t-test). n.s, not significant; *p < 0.05, **p < 0.01, ***p < 0.001.


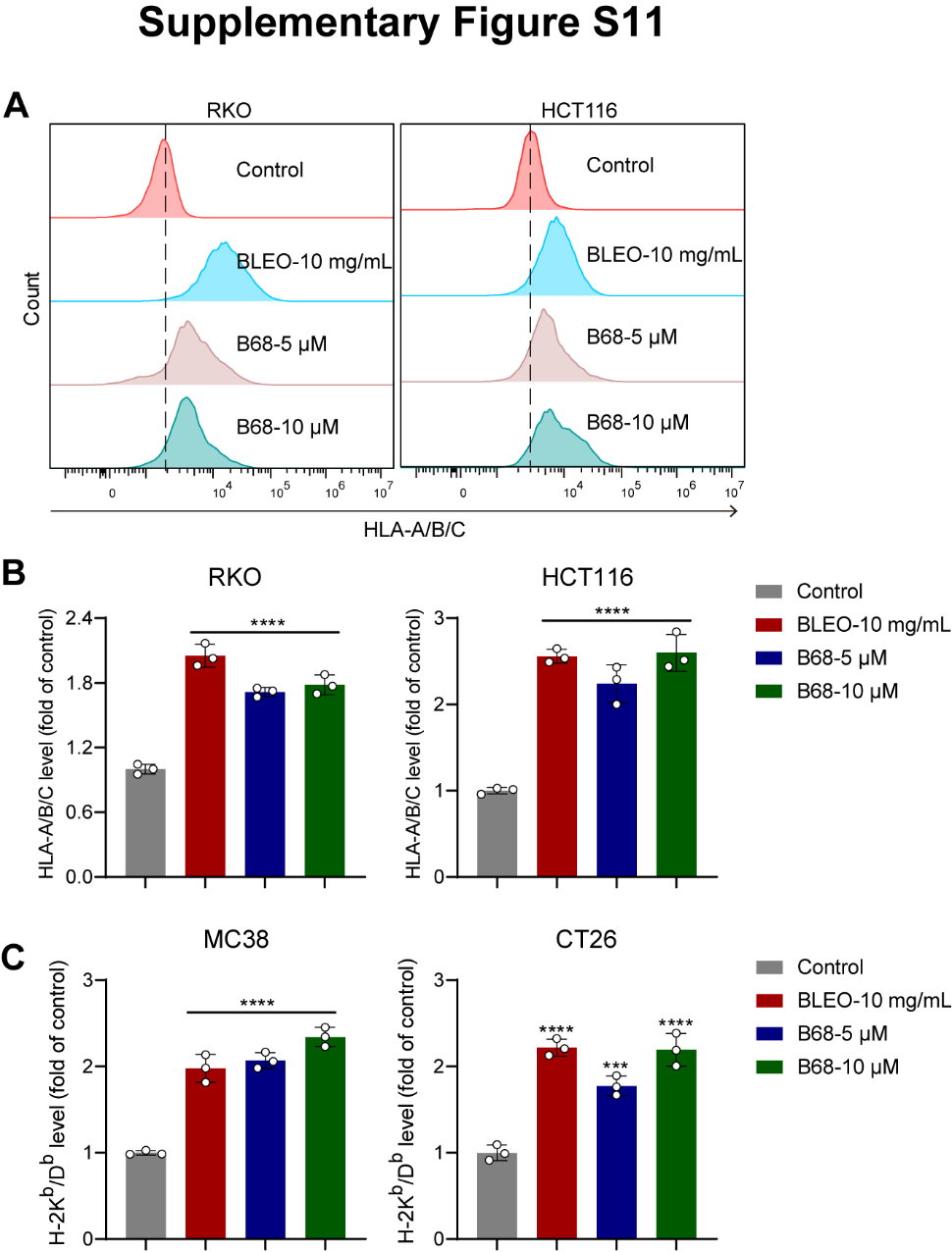


**Figure S11. B68-induced senescent cancer cells efficiently activate DCs**

(A) Flow cytometry analysis of HLA-A/B/C expression in senescent cells (RKO and HCT116) induced by B68. (B) Quantitative results of the expression of HLA-A/B/C in RKO and HCT116 cells induced by B68 (n = 3, error bars represent SEM, mean ± SEM, one-way ANOVA, compared with control). (C) Quantitative results of the expression of H-2K^b^/D^b^ in MC38 and CT26 cells induced by B68 (n = 3, error bars represent SEM, mean ± SEM, one-way ANOVA, compared with control). ***p < 0.001, ****p < 0.0001.


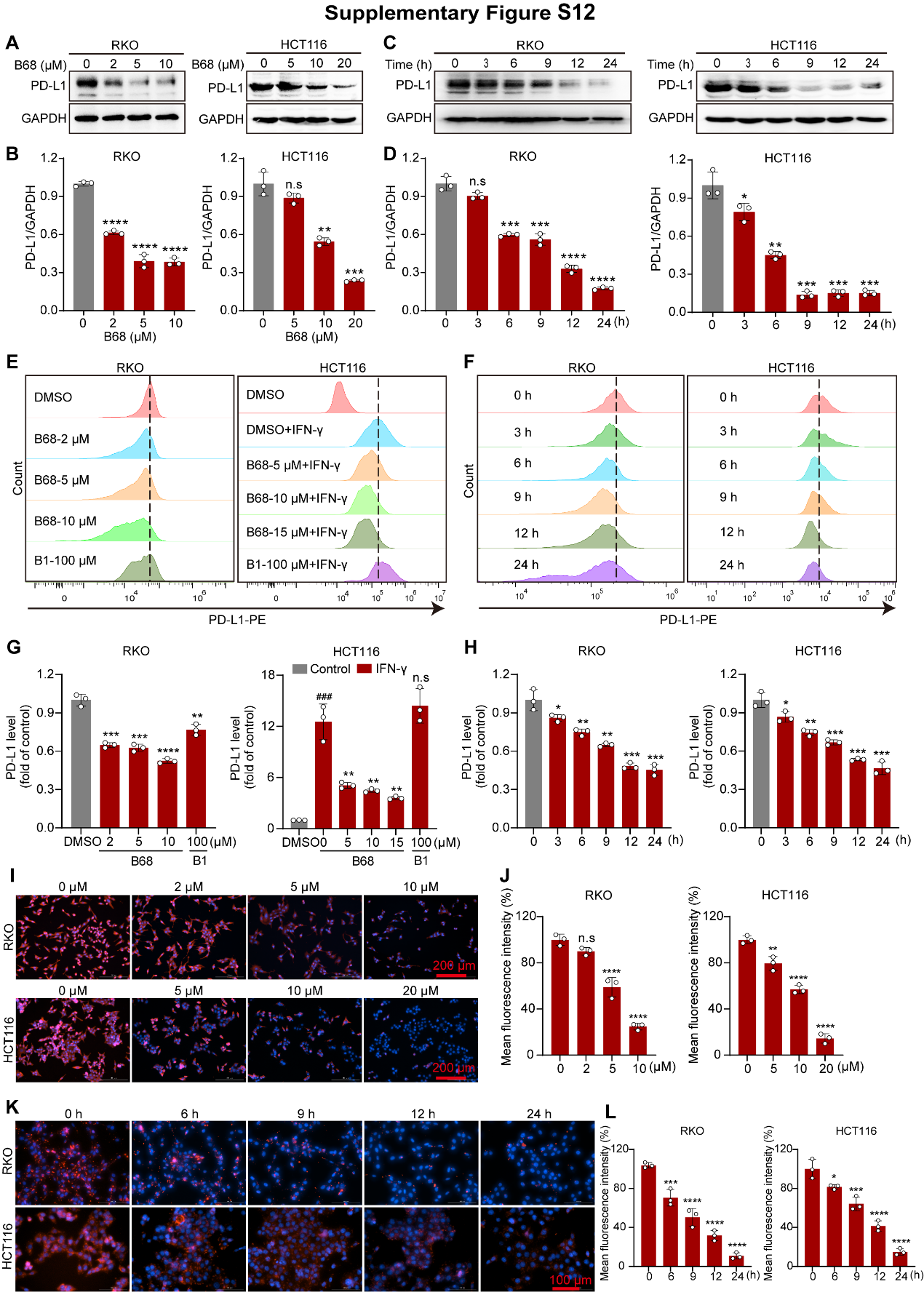


**Figure S12. B68 promotes the degradation of PD-L1 in colorectal cancer cells**

(A) RKO and HCT116 cells were treated with different concentrations of B68 for 24 h. Total PD-L1 protein expression was detected by immunoblotting (IB), and the quantitative results of PD-L1 protein expression are shown below (B) (n = 3, error bars represent SEM, mean ± SEM, Student's t-test). (C) RKO and HCT116 cells were treated with the berberine derivative B68 (10 µM or 20 µM) for the indicated times to detect total PD-L1 protein expression, and the quantitative results of PD-L1 protein expression are shown below (D) (n = 3, error bars represent SEM, mean ± SEM, Student’s t-test). (E, F) RKO and HCT116 cells were treated with different concentrations of B68 or B68 for 24 h (10 µM or 20 µM) for the indicated times, and flow cytometry was performed to detect the expression of PD-L1 on the cell membrane (the stimulation of the proinflammatory factor IFN-γ at 50 ng/mL induced the upregulation of PD-L1 expression), and the quantitative results of the PD-L1 protein are shown below (G, H) (n = 3, error bars represent SEM, mean ± SEM, Student’s t-test). (I, K) The expression of PD-L1 on the membranes of RKO and HCT116 cells treated with B68 (at different concentrations or for different durations) was detected by immunofluorescence, and the nuclei were stained with Hoechst. Scale bars, 100 µm or 200 µm. PD-L1 expression was calculated based on the intensity of red fluorescence. The quantitative results of immunofluorescence staining are shown on the right (J, L) (n = 3, error bars represent SEM, mean ± SEM, one-way ANOVA). n.s, not significant; *p < 0.05, **p < 0.01, ***p < 0.001, ****p < 0.0001.


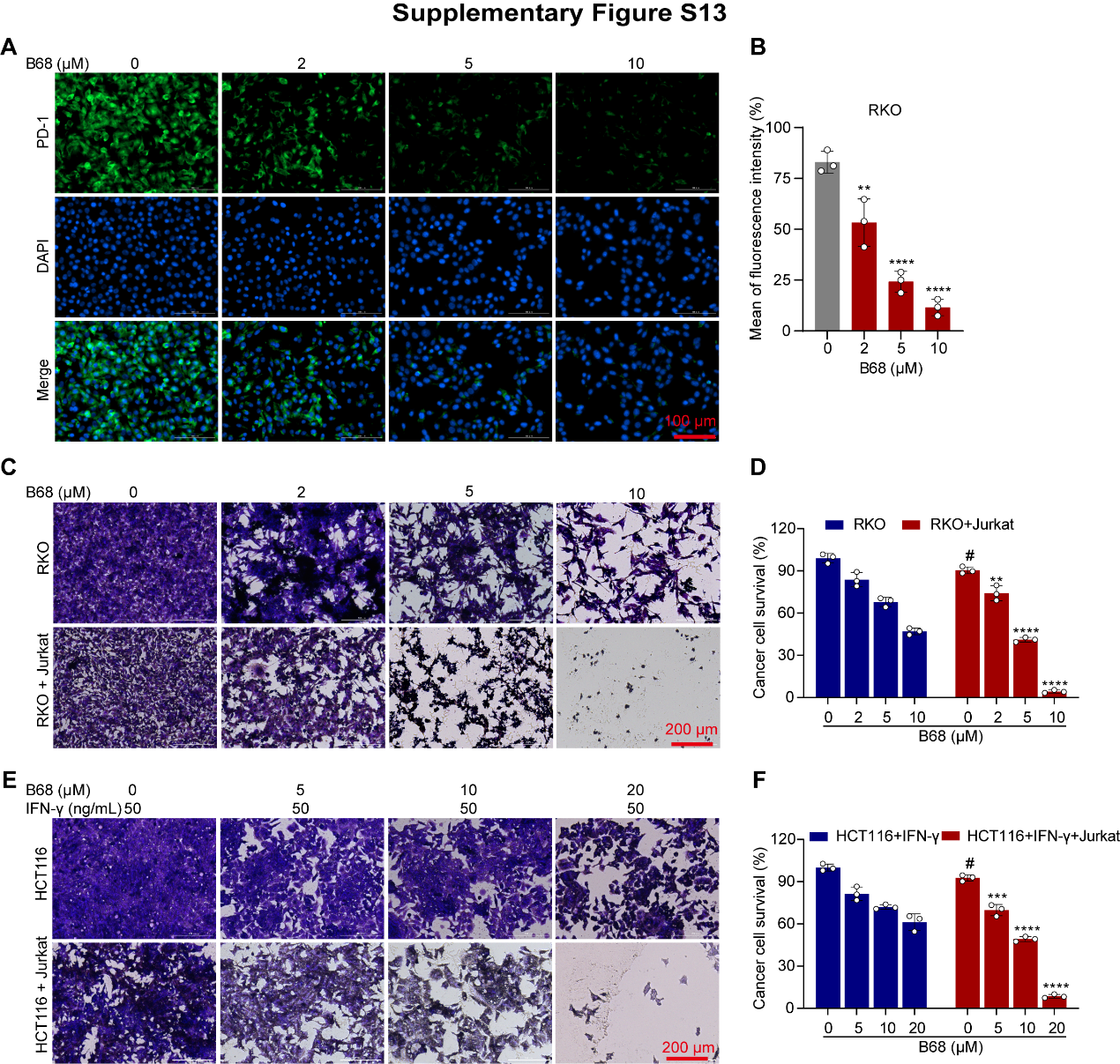


**Figure S13. B68 enhances the cytotoxicity of T cells**

(A) PD-L1/PD-1 binding assay in B68-treated RKO cells. The cell nuclei were stained with Hoechst, and (B) quantitative analysis of PD-1 (green fluorescence) is shown on the right, scale bar, 100 µm. (n = 3, error bars represent SEM, mean ± SEM, one-way ANOVA). (C-F) Jurkat cells stably transfected with PD-1 were cocultured with B68-treated RKO cells or HCT116 cells, and surviving tumor cells were detected using crystal violet staining. The intensities of surviving cells are shown on the right; #p < 0.05, compared with the RKO and HCT116 DMSO groups; **p < 0.01, ***p < 0.001, ****p < 0.0001 compared with the RKO + T cell or HCT116 + T cell DMSO groups (n = 3, error bars represent SEM, mean ± SEM, Student's t-test). n.s, not significant; **p < 0.01, ***p < 0.001, ****p < 0.0001.

**
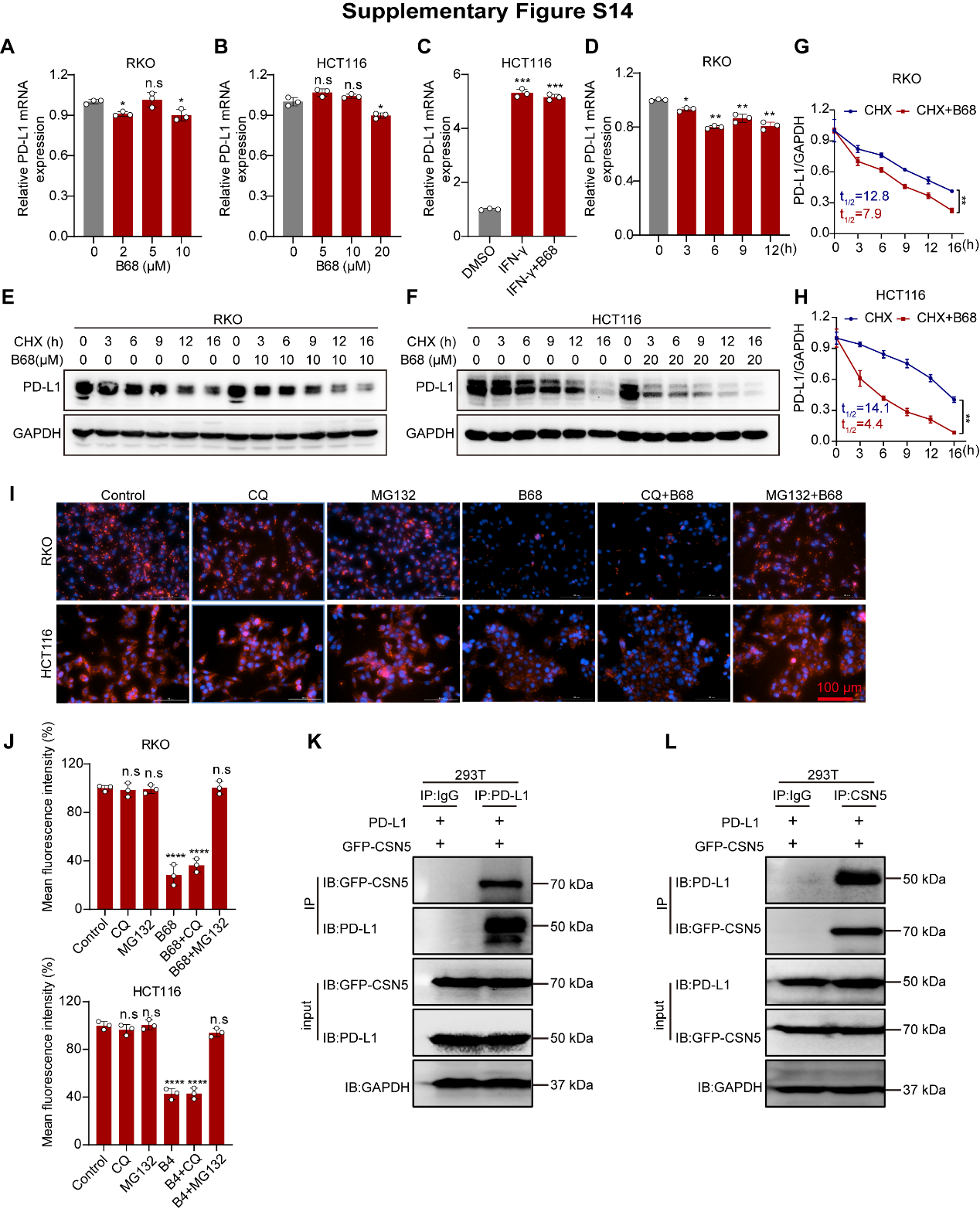
**

**Figure S14. B68 degrades PD-L1 via the ubiquitin proteasome pathway**

(A-D) Quantitative RT-PCR analysis of PD-L1 mRNA levels in RKO and HCT116 cells treated with different concentrations of B68 or RKO cells treated with B68 (10 µM) for the indicated times and B68 (20 µM)- and IFN-γ (50 ng/mL)-treated HCT116 cells (n = 3, error bars represent SEM, mean ± SEM, Student's t-test). (E, F) Immunoblotting was used to detect PD-L1 abundance in RKO and HCT116 cells treated with DMSO or B68 for the indicated time periods in the presence of CHX (50 mg/mL), and (E, F) quantification of PD-L1 intensity is shown in S13 (G) and (H) (n = 3, error bars represent SEM, mean ± SEM, Student’s t-test). (I) Immunofluorescence assay to detect the expression of PD-L1 on the cell membrane of RKO and HCT116 cells treated with B68 in combination with proteasome inhibitor MG132, chloroquine, lysosome inhibitor bafilomycin A1, or autophagy inhibitor 3-methyladenine (nuclei were stained with Hoechst, scale bar, 100 µm). (J) Quantification of PD-L1 expression in the cell membrane following treatment of RKO and HCT116 cells with B68 in combination with the proteasome inhibitor MG132, chloroquine, the lysosomal inhibitor bilomycin A1 or the autophagy inhibitor 3-methyladenine (n = 3, error bars represent SEM, mean ± SEM, one-way ANOVA). (K, L) 293T cells transfected with the PD-L1 plasmid and the CSN5 plasmid were collected for immunoprecipitation (IP) and IB analysis. n.s, not significant; *p < 0.05, **p < 0.01, ***p < 0.001, ****p < 0.0001.


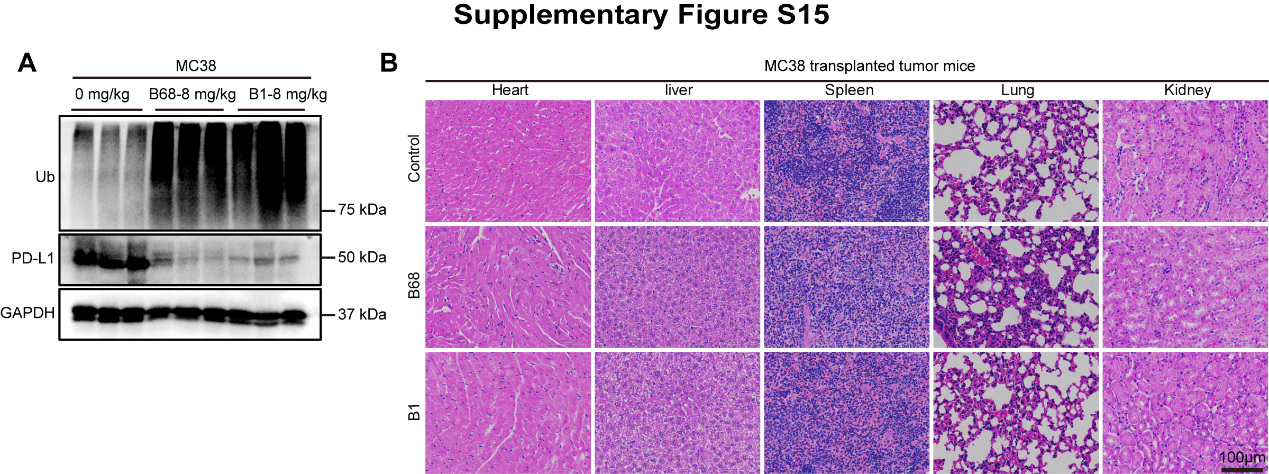


**Figure S1****5. B68 can promote the ubiquitination-mediated degradation of PD-L1 and has no significant effect on the major organs of C57BL/6J mice.**

(A) The levels of ubiquitination and PD-L1 in the tumor tissues of MC38 subcutaneous tumor-bearing mice treated with B68 (8 mg/kg) or B1 (8 mg/kg) were determined via Western blotting. (B) H&E staining revealed that B68 has no significant toxic effects on C57BL/6J mice.


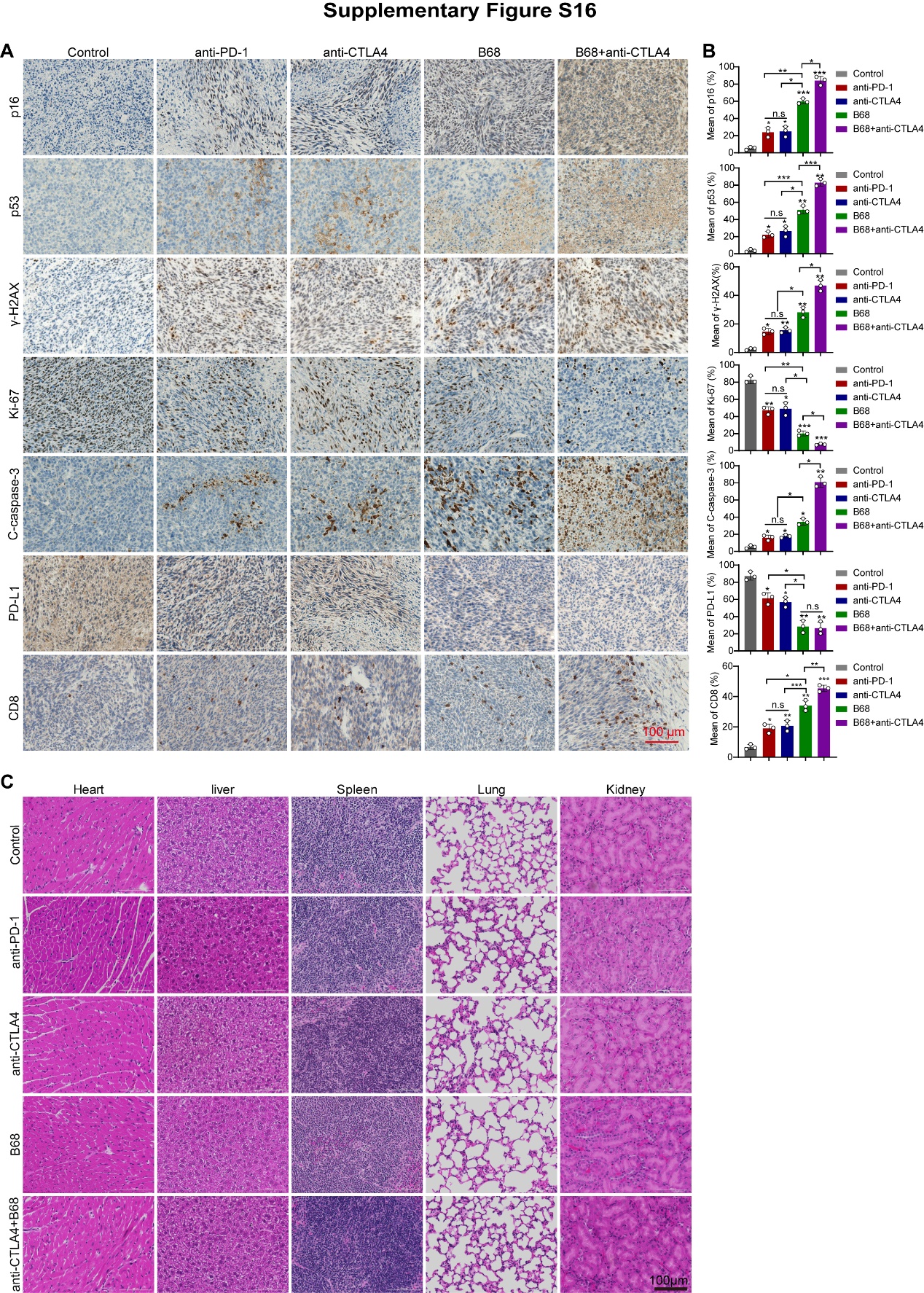


**Figure S16. B68 combined with CTLA4 antibody enhances antitumor effects**

(A, B) Representative immunohistochemical (IHC) staining results for PD-L1, cleaved caspase 3, ki-67, p16, p53, CD8 and γ-H2AX in tumor tissues and quantitative results of immunohistochemical analysis are shown on the right. Scale bar, 100 µm. (n=3, error bars represent SEM, mean ± SEM, Student’s t-test). (C) Representative HE staining results for the heart, lung, liver, spleen, and kidney in the PBS, anti-PD-L1, anti-CTLA4, B68, and B68 + anti-CTLA4 groups. n.s, not significant; *p < 0.05, **p < 0.01, ***p < 0.001.

**Table S1. Antibodies used in this study**

| Antibody | Source | Catalog No. |
| --- | --- | --- |
| Anti-PD-L1  Anti-PD-L1 | Abcam  Proteintech | ab213480  66248-1-Ig |
| Anti-BMI1  Anti-CSN5  Anti-p53  Anti-p21  Anti-p21  Anti-p53 | Proteintech  Proteintech  Santa Cruz  Santa Cruz  Cell Signaling Technology  Cell Signaling Technology | 10832-1-AP  27511-1-AP  Sc-126  Sc-136020  37543  48818 |
| Anti-Ubiquitin  Anti-mouse PD-1 (CD279)  Anti-mouseCTLA4 (CD152)  Anti-GAPDH  Anti-mouse CD80  Anti-mouse CD86  Anti-mouse MHC ClassⅡ(I-A/I-E)  Anti-human HLA-A/B/C  Mouse H-2K^b^/D^b^  PE anti-human CD274  PE anti-mouse CD274  PE anti-mouse CD25  Alexa Fluor® 700 anti-human/mouse  Granzyme B Recombinant Antibody  Anti-Mouse CD4, PE-Cy7  Anti-Mouse Ly-6G(Gr-1), FITC  Anti-Mouse Foxp3, APC  Anti-Mouse CD3ε, APC-Cy7  Anti-Mouse CD8α, PerCP-Cy5.5  Anti-Human/Mouse CD11b, mFluor 450  GM-CSF  IL4 | Abcam  Invivogen  Invivogen  Proteintech  Biolegend  Biolegend  Multi Sciences  invitrogen  Biolegend  Biolegend  Biolegend  Biolegend  Biolegend  Multi Sciences  Multi Sciences  Multi Sciences  Multi Sciences  Multi Sciences  Multi Sciences  PeproTech  PeproTech | ab7245  BE0146  BP0032  60004-1-Ig  104714  159202  70-F21ⅡAE01-100  2492123  114617  329706  124307  101904  372222  70-F2100405/2-100  70-F21LY6G01-100  70-F21FP303-100  70-F21003A06-100  70-F2100804-100  70-F41011b07-100  315-03  214-14 |

**Table S2. Primers for quantitative real-time PCR analysis**

| Name | Primers: forward primers (F), reverse primers (R) |
| --- | --- |
| PD-L1 (H) | Forward: 5′-TCACTTGGTAATTCTGGGAGC-3′ |
|  | Reverse: 5′-CTTTGAGTTTGTATCTTGGATGCC-3′ |
| p16 (H) | Forward: 5’-ACCAGAGGCAGTAACCATGC-3’ |
|  | Reverse: 5’-TGCCTGCTTCTACAAACCCA-3’ |
| p21 (H) | Forward: 5’-GTCAAATCCTCCCCTTCCTGG-3’ |
|  | Reverse: 5’-AGTCGAAGTTCCATCGCTCA-3’ |
| BCL2 (H) | Forward: 5′-GAGGATTGTGGCCTTCTTTG-3′ |
|  | Reverse: 5′-ACAGTTCCACAAAGGCATCC-3′ |
| BAX (H) | Forward: 5′-TTTGCTTCAGGGTTTCATCC-3′ |
|  | Reverse: 5′-CATGTGAAGTTGCCGTCAGA-3′ |
| IL6 (H) | Forward: 5’-TACCCCCAGGAGAAGATTCC-3’ |
|  | Reverse: 5’-TTTTCTGCCAGTGCCTCTTT-3’ |
| CXCL8 (H) | Forward: 5’-GTGCAGTTTTGCCAAGGAGT-3’ |
|  | Reverse: 5’-CTCTGCACCCAGTTTTCCTT-3’ |
| CXCL3 (H) | Forward: 5’-GCAGGGAATTCACCTCAAGA-3’ |
|  | Reverse: 5’-GGTGCTCCCCTTGTTCAGTA-3’ |
| GM-CSF (H) | Forward: 5′-CCCCAGTCACCTGCTGTTAT-3′ |
|  | Reverse: 5′-TGGAATCCTGAACCCACTTC-3′ |
| Areg (H) | Forward: 5′-CTCTGGGAAGCGTGAACCAT-3′ |
|  | Reverse: 5′-GTAGTCATAGTCGGCTCCCG-3′ |
| Spink1 (H) | Forward: 5′-TGGGAAGAGAGGCCAAATGTT-3′ |
|  | Reverse: 5′-AGTCTGGCGTTTCCGATTTT-3′ |
| Nlrc5 (M) | Forward: 5’-TCAGGAAGTTTGACCTCTCAGG-3’ |
|  | Reverse: 5’-GTTATTCTCTGCCAAGCTGACC-3’ |
| B2m (M) | Forward: 5’-GCCGAACATACTGAACTGCT-3’ |
|  | Reverse: 5’-GCCATACTGGCATGCTTAAC-3’ |
| Tap2 (M) | Forward: 5’-CGGTGCTAAAGGAGATCCAG-3’ |
|  | Reverse: 5’-CCATCACCCTCCGTATGACT-3’ |
| p53 (M) | Forward: 5’-TCTGGGACAGCCAAGTCTGT-3’ |
|  | Reverse: 5’-GGAGTCTTCCAGTGTGATGA-3’ |
| p21 (M) | Forward: 5’-AGGGTGTCACCGAGAGGTTTA-3’ |
|  | Reverse: 5’-GGGATGCACTGGGTGTTCTT-3’ |
| p16 (M) | Forward: 5’-TCAAAAAGGTATGGTTCCGACC-3’ |
|  | Reverse: 5’-GCAGGCGACGGTAATCCAG-3’ |
| GAPDH (M) | Forward: 5’-GTTGTCTCCTGCGACTTCA-3’ |
|  | Reverse: 5’ GGTGGTCCAGGGTTTCTTA-3’ |
| GAPDH (H) | Forward: 5’-GCATTGCCCTCAACGACCAC-3’ |
|  | Reverse: 5’-CCACCACCCTGTTGCTGTAG-3’ |
| BMI1 (H) | Forward: 5′-CTGGTTGCCCATTGACAGC-3′ |
|  | Reverse: 5′-CAGAAAATGAATGCGAGCCA-3′ |
| S100A9 (H) | Forward: 5’-GCACCCAGACACCCTGAACCA-3’ |
|  | Reverse: 5’-TGTGTCCAGGTCCTCCATGATC-3’ |
| YBOX1 (H) | Forward: 5’-ATGCCGGCTTACCATCTCTACC-3’ |
|  | Reverse: 5’-TCAACGGGCAAAAAGCAAGC-3’ |
| UBAP2L (H) | Forward: 5′-ATTCGCCTCACTCTCCACAC-3′ |
|  | Reverse: 5′-TACCACCACACAACACAGCA-3′ |

**Table S3. Sequences of the siRNAs/sgRNAs used in this study**

| Name | SiRNAs sequence |
| --- | --- |
| si-BMI1#1 | 5′-CCAAGAUAUUGUAUACAAATT-3′ |
| si-BMI1#2 | 5′-GGAGGAACCUUUAAAGGAUUA-3′ |
| si-S100A9#1 | 5′-CCAAAUAAAGUCUCUUCCUTT-3′ |
| si-S100A9#2 | 5′-CUGUUAUGUCAAACUGUCUTT-3′ |
| si-YBOX1#1 | 5′-GUUCAAUGUAAGGAACGGAU-3′ |
| si-YBOX1#2  si-UBAP2L#1  si-UBAP2L#2  sg-BMI1#1  sg-BMI1#2 | 5′-GACGGCAAUGAAGAAGAUAA-3′  5′-GCCUGUCCUUUCUGAUUAUTT-3’  5′-GCCACAAGUAUAUGGUUAUTT-3’  5′-ATTGTATACAAATTAGTTCC-3’  5′-GCAGCTCATCCTTCTGCTGA-3’ |
